# A colorimetric hydroxy naphthol blue based loop-mediated isothermal amplification detection assay targeting the β-tubulin locus of *Sarocladium oryzae* infecting rice seed

**DOI:** 10.3389/fpls.2022.1077328

**Published:** 2022-11-21

**Authors:** R. Logeshwari, C. Gopalakrishnan, A. Kamalakannan, J. Ramalingam, R. Saraswathi

**Affiliations:** ^1^ Department of Plant Pathology, Tamil Nadu Agricultural University, Coimbatore, India; ^2^ Department of Plant Biotechnology, Tamil Nadu Agricultural University, Coimbatore, India; ^3^ Department of Plant Genetic Resources, Tamil Nadu Agricultural University, Coimbatore, India

**Keywords:** *Sarocladium oryzae*, seed, β-tubulin, HNB dye, Loop-mediated isothermal amplification, *Oryza sativa*

## Abstract

Sarocladium oryzae is a widely prevalent seed-borne pathogen of rice. The development of a rapid and on-site detection method for *S. oryzae* is therefore important to ensure the health of rice seeds. Loop-mediated isothermal amplification (LAMP) is ideal for field-level diagnosis since it offers quick, high-specific amplification of target template sequences at a single temperature. We designed primers based on the β-tubulin region of *S. oryzae*. The LAMP technique devised was extremely sensitive, detecting the presence of the *S. oryzae* template at concentrations as low as 10 fg in 30 minutes at 65°C. The assay specificity was confirmed by performing the experiment with genomic DNA isolated from 22 different phytopathogens. Through the addition of hydroxy naphthol blue in the reaction process prior to amplification, a colour shift from violet to deep sky blue was seen in the vicinity of the target pathogen only. Finally, the LAMP assay was validated using live infected tissues, weeds and different varieties of seeds collected from different locations in Tamil Nadu, India. If developed into a detection kit, the LAMP assay developed in this study has potential applications in seed health laboratories, plant quarantine stations, and on-site diagnosis of *S. oryzae* in seeds and plants.

## 1 Introduction

Rice (*Oryza sativa* L.) is a substantial food crop that contributes significantly to the global economy. It is the primary food crop for almost two-thirds of the world’s population, coming in third place globally behind maize and wheat in terms of production (Abbade, 2021). According to a comprehensive survey made by the International Food Policy Research Institute, rice production will need to increase by 38% by 2030 to feed the world’s growing population, yet available arable land is being lost to housing and industrialization (Zhang et al., 2019). Even if the annual rise in rice output is high, there are still several production barriers, such as pests and diseases. Diseases have caused a significant loss in rice output, amounting up to 80% of the entire rice crop in severe cases (Nasruddin and Amin, 2013). The maintenance of rice seed health is crucial to secure food availability and achieving significant export because the number of organisms that threaten rice cultivation is abundant (Teng, 1994; Reddy and Sathyanarayana, 2001; Dossou and Silue, 2018).

A significant loss in quality and yield can be caused by seed-borne pathogens, which may also serve as an unnoticed source for the spread of pathogens (Tiwari, 2016). Early detection of seed-borne fungal infections is critical to minimize uncontrolled pathogen propagation *via* long-distance exchange of such material, as well as to avoid economic losses and superfluous application of fungicide (Mancini et al., 2016). Rice Sheath Rot incited by *Sarocladium oryzae*, one of the most damaging rice seed-borne fungal diseases, resulting in significant economic losses in rice-growing countries. The pathogen was first reported in 1922 in Taiwan (Mathur, 1981; Mew and Gonzales, 2002; Ayyadurai et al., 2005; Bigirimana et al., 2015). This pathogen thrives well in rainfed rice fields and is prevalent in lowland and medium-land habitats (Pearce et al., 2001; Sarangi and Islam, 2019). The losses were in the form of qualitative as well as quantitative *i.e.*, loss of yield including discoloration of grain that renders it unfit for export (Gopalakrishnan et al., 2010; Zhang et al., 2019).

The detection of seed-borne *S. oryzae* is vital during seed certification and allows for the implementation of early containment and control measures. Early detection of RSR is crucial for regulating the spread of the disease, minimizing direct and indirect economic losses like the excessive application of fungicides, and preventing excessive pathogen spread. The common method used for diagnosis of *S. oryzae* in the laboratory is standard blotter tests. However, it requires laboratory facilities and incubation for several days. Nucleic acid-based approaches have become widely used to identify pathogens more quickly. To address this, LAMP is an isothermal nucleic acid amplification technology that has several benefits in diagnostic research. Due to the lack of thermal cycling and the tendency of the enzymes to replicate more quickly than PCR, reactions can be carried out using portable, battery-operated platforms. The LAMP test utilizes four to six oligonucleotide primers that were intended to recognize a total of six distinct regions of the target gene, and two loop primers positioned in the loop region might speed up the amplification (Notomi et al., 2000; Zhang et al., 2016) together with the strand displacement activity of *Bst* DNA polymerase, which may amplify precise DNA sequences with great specificity (Wastling et al., 2000).

In this investigation, LAMP assay was established for the quick, facile, specific and highly sensitive detection of *S. oryzae* in rice seeds. With these assays, *S. oryzae* may be rapidly detected in infected rice tissues in the field as well as in seeds.

## 2 Materials and method

### 2.1 Sample collection, isolation of pathogen and DNA extraction

Fifty *S. oryzae* isolates were isolated from rice grain samples which were randomly collected from farmer’s field, storage godowns and research centres from different locations of Tamil Nadu, India, labeled appropriately and brought to the laboratory for further studies. Each isolates were incubated at 27 ± 2°C for 10- 20 days on potato dextrose agar (PDA) media. For extracting DNA from fungal mycelia, cultures were grown in a 250 ml conical flask with 100 ml of potato dextrose broth that had been autoclaved at 121°C and 15 psi. The fungal mycelial discs (4 no.) were then inoculated into flasks and incubated at 27 ± 2°C for 7-10 days. The mycelial mat was taken from the broth and dried on filter paper. The modified CTAB method was used to extract total genomic DNA (Doyle, 1991). A Nano-drop Spectrophotometer (ND-1000) was used to measure the concentration and purity of each genomic DNA, and the quality of all extracted DNA was determined by measuring its absorbance at 260 and 280 nm. Agarose gel electrophoresis was done to confirm DNA concentrations. On an agarose gel with a 1.2% (w/v) concentration and 0.1 µg/mL of ethidium bromide, ten microliters of purified DNA were run. The Biorad Gel Doc was used to visualize the DNA. The DNA was kept at -20°C for further use.

### 2.2 PCR detection and sequencing

PCR analysis was carried out in a 25 µL reaction mixture containing 1 µL of genomic DNA, 12.5 µL of 2X Master mix (RR310 EmeraldAmp), and 2.5 µL of both forward and reverse primer (ITS 1F and ITS 4R). Nuclease-free water was added to bring the total amount to 25 µL. DNA amplification was accomplished in an Eppendorf thermocycler (Germany) under the following cycling conditions: primary denaturation at 95°C for 2 min; 35 cycles of denaturation at 95°C for 30 s; annealing at 55°C for 30 s; elongation at 72°C for 60 s and terminal elongation 72°C for 10 min. The DNA bands were separated by gel electrophoresis in a 1.2% agarose (Himedia) gel stained with 0.1 µg/mL of ethidium bromide. The UV gel documentation system was used to visualize the DNA bands resolved on agarose gel. By contrasting the amplicons with a 100 bp DNA ladder (3422A Takara) on an agarose gel, the amplicons sizes were estimated. The amplified PCR product was sequenced at Biokart India Pvt. Ltd. (Bangalore, India) by Sanger’s dideoxy method. The sequences were compared with other ITS region of *S. oryzae* available at NCBI database to assess for their variability (**Supplementary Table 1**).

### 2.3 Primer design and *in silico* validation

**Figure 1:**
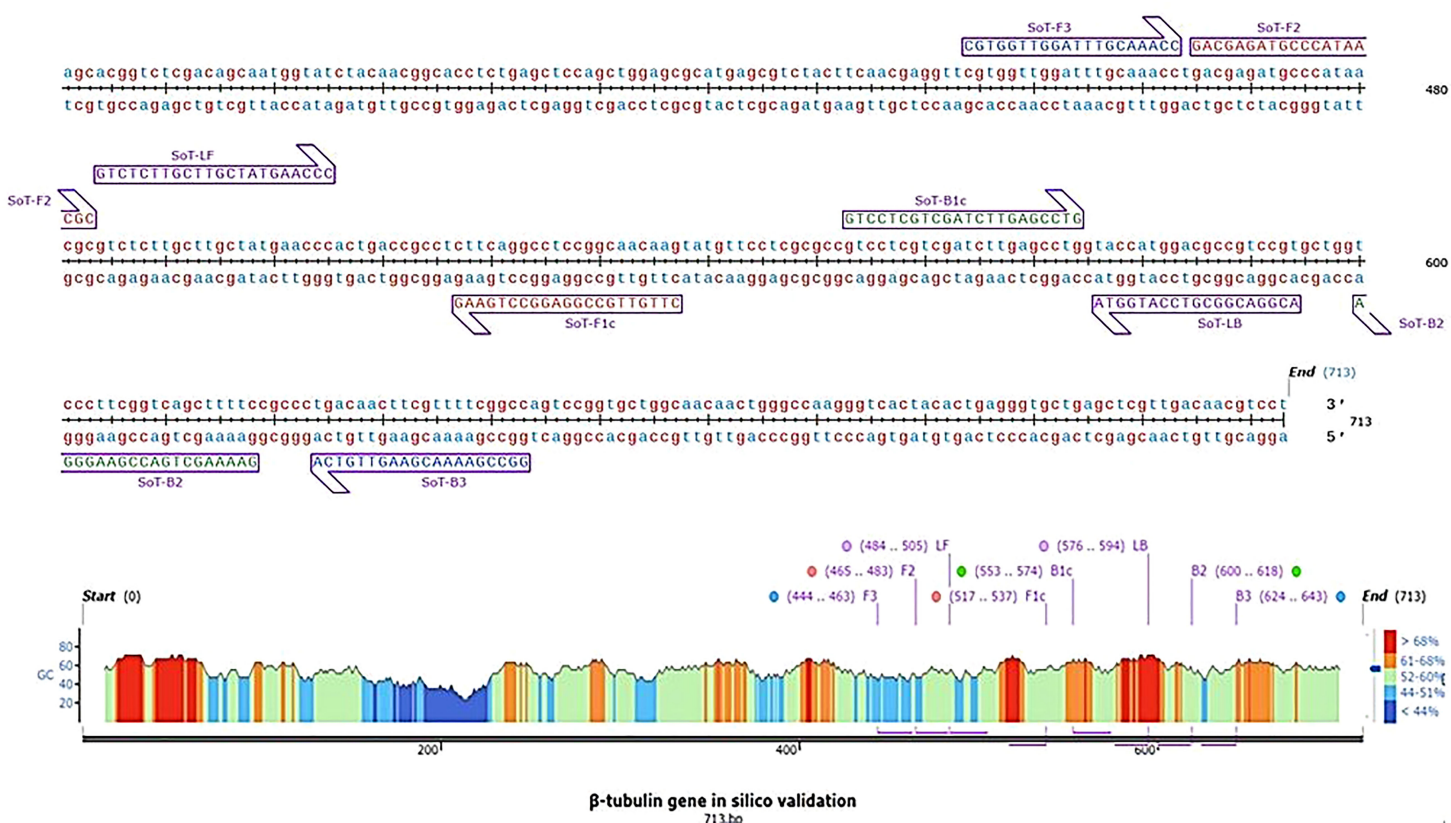
Diagrammatic illustration of *in silico* hybridization of LAMP primers positioned within the β-tubulin (713 bp) domain of the pathogen *Sarocladium oryzae* visualized using SnapGene viewer^®^ 6.1.1. Outer primers: F3 and B3 (blue), loop primers: LF and LB (purple), inner primers: F2 and F1c (Red) and B1c and B2 (green).

The software tool PRIMER EXPLORER V5 (http://primerexplorer.jp/e/) which is accessible on the Eiken Genome website was used to design LAMP primers. A critical part in LAMP primer designing is the selection of a potential target gene with high nucleotide variability among related species. LAMP primers were designed by utilizing the β-tubulin gene of *S. oryzae*. A set of six primers was designed by using the sequences retrieved from NCBI GenBank (Accession number MZ614962.1). Each parameter was set to its default value. The primer sequences utilized in this experiment are furnished in **Table 1**. **Figure 1** depicts LAMP primer’s structure and its complement target DNA used in this work. In addition, β-Tubulin sequences of *S. oryzae* strain Sor-3 (MZ614962.1), *S. oryzae* strain Sor-2 (MZ614961.1),

**Table 1:** Sequence of oligonucleotide primer sets used for amplification of target sequence in β-tubulin gene in this study.

Primer name	Primer type	Sequence 5’→3’	Primer length (nt)	GC (%)	GC clamp	3’ΔG
Tub-F3	Forward outer	CGTGGTTGGATTTGCAAACC	20	50	2	-4.77
Tub-B3	Backward outer	GGCCGAAAACGAAGTTGTCA	20	50	2	-4.58
Tub-FIP (F1c-F2)	Forward inner	CTTGTTGCCGGAGGCCTGAAG-GACGAGATGCCCATAACGC	40	60	3	-4.79
Tub-BIP(B1c-B2)	Backward inner	GTCCTCGTCGATCTTGAGCCTG-GAAAAGCTGACCGAAGGGA	41	56	3	-5.64
Tub-LF	Forward loop	GGGTTCATAGCAAGCAAGAGAC	22	50	3	-4.59
Tub-LB	Backward loop	TACCATGGACGCCGTCCGT	19	63	3	-6.18

**Figure 2:**
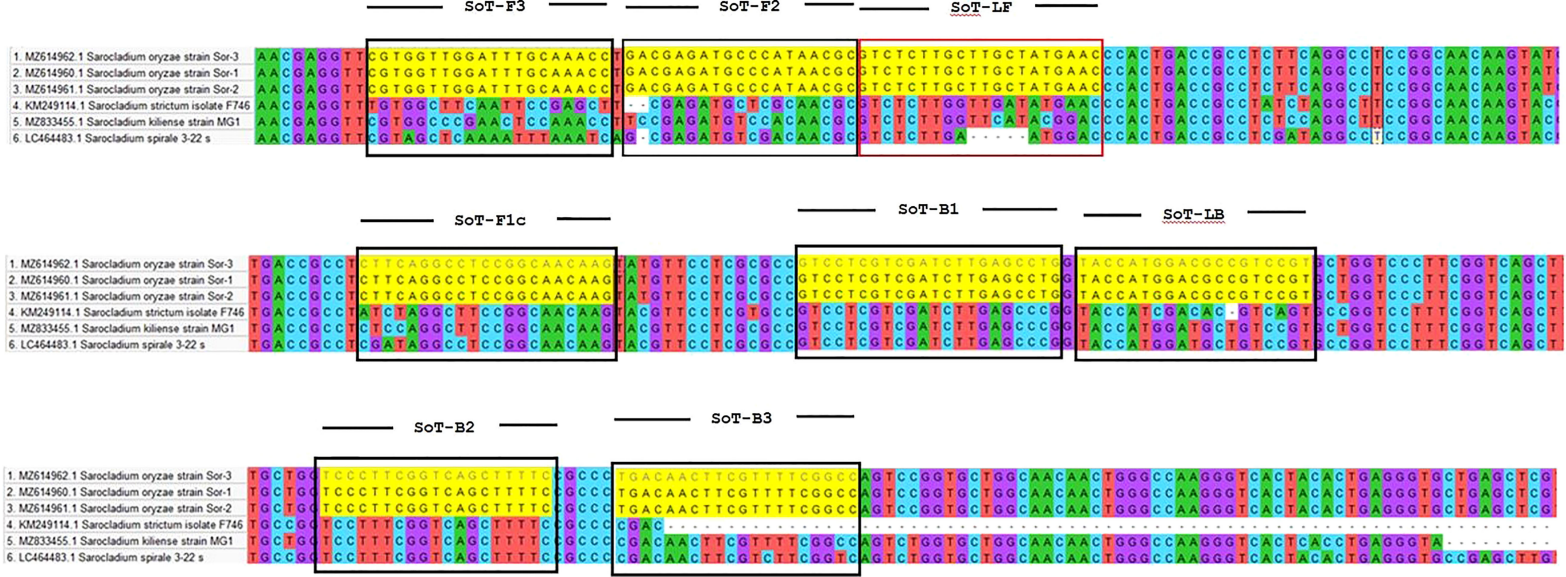
Comparison of the β-tubulin region of *S. oryzae* with other *Sarocladium* spp.

*S. oryzae* strain Sor-1 (MZ614960.1), *S. strictum* (KM249114.1), *S. kiliense* (MZ833455.1) and *S. spirale* (LC464483.1) were acquired from the NCBI website (http://www.ncbi.nlm.nih.gov) to verify the specificity of primers against other three *Sarocladium* spp. (**Figure 2**). Oligo Evaluator™ (Sigma Aldrich) was employed to verify developed primers for secondary structure formation (hairpins, palindromes, repetitions, dimers, cross dimers and runs), primer dimerization (primer-dimer), % GC and GC clamp (https://www.sigmaaldrich.com) (Zaczek-Moczydłowska et al., 2020). The specificity of each primer was confirmed by comparing the primer sequences with the NCBI GenBank nucleotide and genome databases by employing the BLASTn programme (Altschul et al., 1990).

### 2.4 LAMP reaction

The LAMP reagents were standardized in various concentrations (**Table 2**) in order to determine the best conditions for the reaction. The final LAMP reactions were accomplished in a 25 µL LAMP reaction mixture that contained 1.5 µL of target DNA (~ 120 ng), 2.5 µL of Thermopol reaction buffer (10x), 1.4 mM of each dNTPs, 0.2 mM of both outer primers, 0.4 mM of both loop primers, 1.4 M of both inner primers, 1.2 M betaine, 8 mM MgSO_4_, 2 U *Bst* polymerase (0.08 U/µL) and 120 µM hydroxyl naphthol blue. Instead of using a DNA template, the nuclease-free water served as the negative control. The reactions were performed in an Eppendorf thermal cycler for 30 min at a constant temperature of 65°C, and then the reaction was ceased by thermal denaturation for 2 min at 80°C.

**Table 2:** Standardization of reagents used in LAMP assay.

S. No.	Reagent	Stock Conc.	Concentration of Tested Reagents/1 µL Reaction Volume				
1.	MgSO_4_ (New England BioLabs)	100 mM	2mM	4 mM	6 mM	**8 mM**	10 mM
2.	dNTP Mix (Genei)	10 mM	0 mM	0.6 mM	0.8 mM	1.2 mM	**1.4 mM**
3.	Betaine (Sigma Aldrich)	5M	0 M	0.6 M	0.8 M	**1.2 M**	1.4 M
4.	*Bst* DNA polymerase Large Fragments (New England BioLabs)	8U	0 U	**2 U**	4 U	6 U	8 U
5.	HNB dye	400 µM	80 µM	100 µM	**120 µM**	148 µM	300 µM

The values (bold) indicated optimal reagents concentrations selected for LAMP assay.

A visual evaluation of the colour was done after termination. The quantity of amplification product as evaluated by 2% agarose gel electrophoresis was used to choose the optimal condition (**Supplementary Figure 1**). Protocols with varying amplification temperature (56-70°C) and amplification time (10-150 min) were investigated to optimize the LAMP assay (**Supplementary Figure 2**).

### 2.5 Sensitivity of LAMP assay

**Figure 3:**
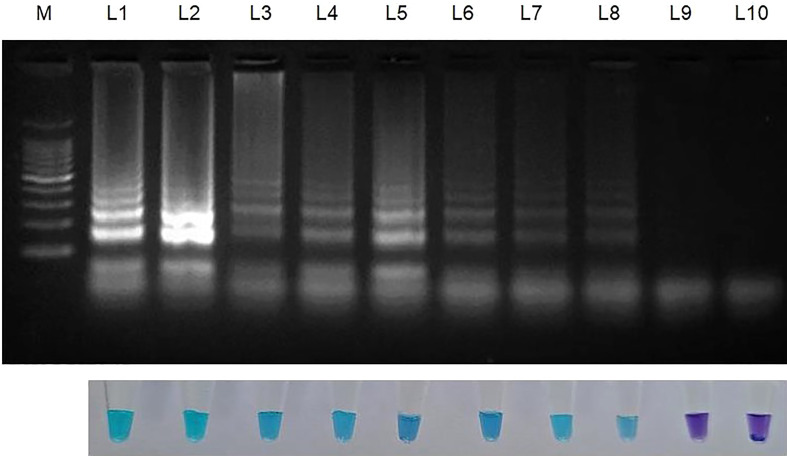
Sensitivity of LAMP assay using ten-fold serially diluted genomic DNA (from 100ng to 1 fg) from *Sarocladium oryzae* isolate OM841519 as template and nuclease free water as negative control. Upper panel in the figure shows agarose gel electrophoresis results whereas lower panel shows reaction tubes for colorimetric detection. M- 100bp Ladder; Lane 1: 100 ng; Lane 2: 10 ng; Lane 3: 1 ng; Lane 4: 100 pg; Lane 5: 10 pg; Lane 6: 1 pg; Lane 7: 100 fg; Lane 8: 10fg; Lane 9: 1 fg; Lane 10: Nuclease free water (Control).

Genomic DNA that had been serially diluted ten times (100 ng/l to 1 fg/l) was used to assess the amplification potency and sensitivity of the LAMP primers. This experiment was carried out thrice (**Table 3**; **Figure 3**).

**Table 3:** Sensitivity of LAMP assay for the detection of *S. oryzae* targeting β-tubulin gene.

S. No.	Concentration of genomic DNA template	LAMP assay
1.	100 ng	+
2.	10 ng	+
3.	1 ng	+
4.	100 pg	+
5.	10 pg	+
6.	1 pg	+
7.	100 fg	+
8.	10 fg	+
9.	1 fg	–

### 2.6 Specificity of LAMP assay

**Figure 4:**
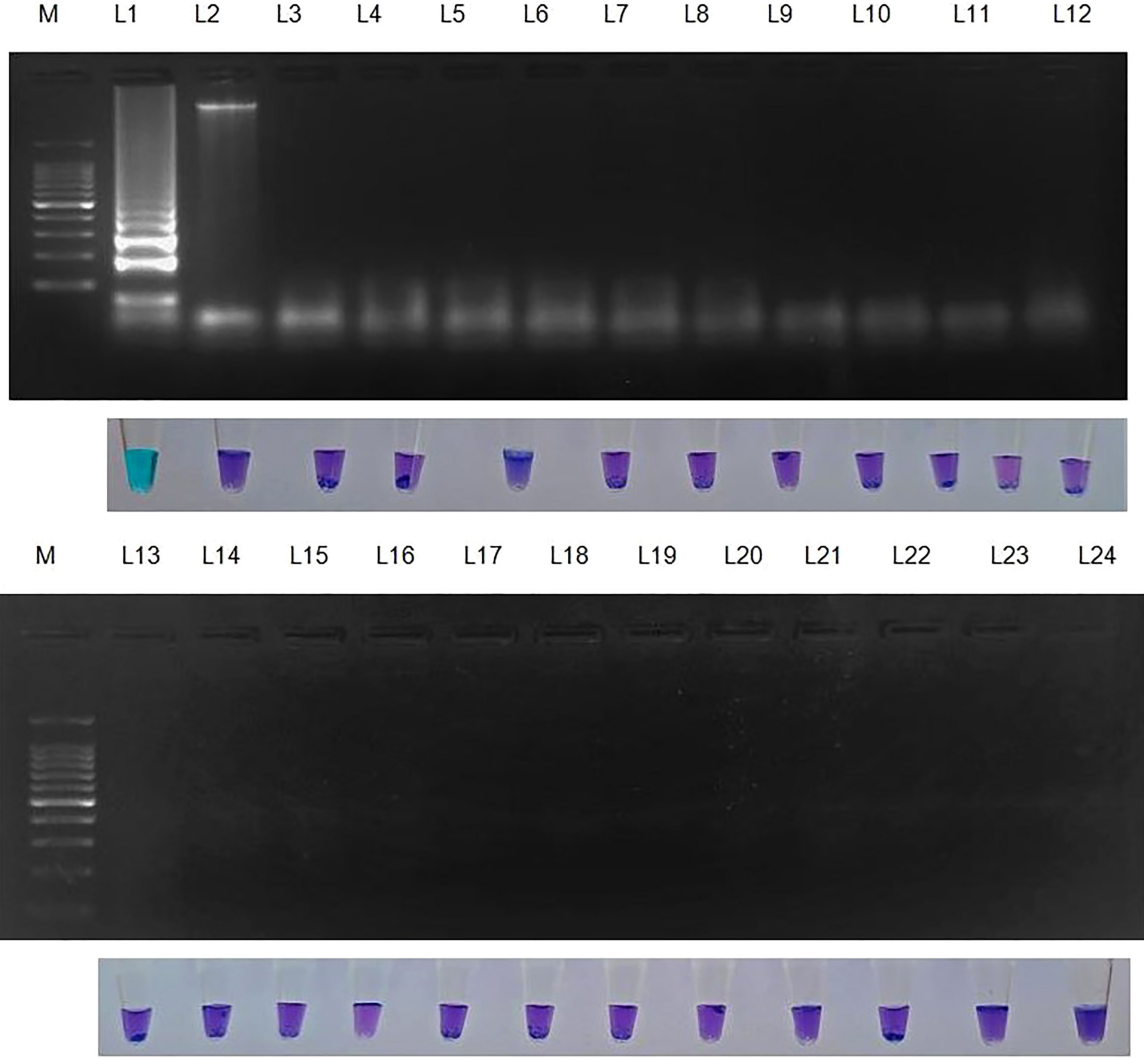
Specificity of LAMP assay using *Sarocladium oryzae* isolate OM841519 as template and nuclease free water as negative control. Upper panel in the figure shows agarose gel electrophoresis results whereas lower panel shows reaction tubes for colorimetric detection. M- 100bp Ladder; Lane 1: *Sarocladium oryzae*; Lane 2: *Sarocladium attenuatum*; Lane3: *Sarocladium strictum;* Lane 4: *Bipolaris oryzae*; Lane 5: *Magnaporthe oryzae*; Lane 6: *Rhizoctonia solani*; Lane 7: *Ustilaginoidea virens*; Lane 8: *Exserohilum rostratum*; Lane 9: *Simplicillium obclavatum*; Lane 10: *Pyricularia grisea*; Lane 11: *Helminthosporium turcicum*; Lane 12: *Macrophomina phaseolina*; Lane 13: *Sclerotium rolfsii*; Lane 14: *Sclerotinia sclerotiorum*; Lane 15: *Colletotrichum gloeosporioides*; Lane 16: *C. scovillea*; Lane 17: *C. truncatum*; Lane 18: *Fusarium oxysporum* f. sp. *cubense*; Lane 19: *Phytophthora infestans*; Lane 20: *Puccinia arachidis*; Lane 21: *Uncinula nector*; Lane 22: *Pantoea dispersa*; Lane 23: *Ralstonia solanacearum*; Lane 24: Nuclease free water (Negative control).

Genomic DNA samples from 22 different phytopathogens (**Table 4**), which were obtained from the Culture Collection Centre, Department of Plant Pathology at Tamil Nadu Agricultural University in Coimbatore, India were tested to ascertain the specificity of the LAMP assay (**Figure 4**).

**Table 4:** Specificity of LAMP assay for the detection of *S. oryzae* targeting β-tubulin gene.

S. No.	Organism	NCBI genbank Accession number	Host	Result
1.	*Sarocladium oryzae*	OM841519	Paddy	+
2.	*Sarocladium attenuatum*	OP099851	Paddy	**-**
3.	*Sarocladium strictum*	OP377063	Paddy	**-**
4.	*Bipolaris oryzae*	MZ995432	Paddy	**-**
5	*Magnaporthe oryzae*	OP303344	Paddy	**-**
6.	*Rhizoctonia solani*	OP412773	Paddy	**-**
7.	*Ustilaginoidea virens*	MZ157263	Paddy	**-**
8.	*Exserohilum rostratum*	OM891473	Paddy	**-**
9.	*Simplicillium obclavatum*	OP501088	Paddy	**-**
10.	*Bipolaris sorokianiana*	OP303316	Wheat	**-**
11.	*Pyricularia grisea*	ON116174	Cumbu	**-**
12.	*Exserohilum turcicum*	ON524816	Maize	**-**
13.	*Macrophomina phaseolina*	ON945550	Groundnut	**-**
14.	*Sclerotium rolfsii*	ON945552	Groundnut	**-**
15.	*Sclerotinia sclerotiorum*	ON025544	Cabbage	**-**
16.	*Colletotrichum gloeosporioides*	ON799265	Mango	**-**
17.	*Colletotrichum scovillea*	ON182070	Chilli	**-**
18.	*Colletotrichum truncatum*	ON182072	Chilli	**-**
19.	*Fusarium oxysporum* f. sp. *cubense*	OM103052	Banana	**-**
20.	*Phytophthora infestans*	ON705720	Potato	**-**
21.	*Puccinia arachidis*	OL437026	Groundnut	**-**
22.	*Uncinula nector*	MK637521	Grapes	**-**
23.	*Pantoea dispersa*	OP378111	Paddy	**-**
24.	Nuclease free water	–	–	–

### 2.7 Validation of LAMP assay with different isolates of *S. oryzae*

The LAMP assay technique developed was validated with different isolates of *S. oryzae* collected from various locations of Tamil Nadu (**Table 5**; **Supplementary Figure 3**). As mentioned earlier, genomic template was extracted using a modified CTAB technique. The LAMP assay was carried out on the DNA of 50 fungal isolates, with nuclease-free water serving as a negative control. The LAMP reaction mixture was incubated at 65°C for 30 min, and then the reaction was ceased by heating it at 80°C for 2 min. The results were visualized by HNB dye, and the experiments were carried out thrice.

**Table 5:** Validation of LAMP assay for the detection of different isolates of *S. oryzae*.

S. No.	Isolate code	Location	Latitude	Longitude	Result
1.	So 1	PBS, Coimbatore	11.0137°N	76.9354°E	Positive
2.	So 2	Thirukovilur	11.9687°N	79.2086°E	Positive
3.	So 3	Vasanthakrishnapuram	12.0455°N	79.2232°E	Positive
4.	So 4	Wetland, Coimbatore	11.0031°N	76.9249°E	Positive
5	So 5	Chidambaram	11.3909°N	79.6662°E	Positive
6.	So 6	Emappur	11.8698°N	79.3702°E	Positive
7.	So 7	Thiruvannamalai	12.2286°N	79.0665°E	Positive
8.	So 8	Kondathur	11.1792°N	79.7079°E	Positive
9.	So 9	Vellimalaipattinam, CBE	10.9779°N	76.7610°E	Positive
10.	So 10	Thiruvennainallur	11.8570°N	79.3682°E	Positive
11.	So 11	Mayiladuthurai	10.9010°N	79.1472°E	Positive
12.	So 12	Kallakurichi	11.7652°N	79.0706°E	Positive
13.	So 13	Sathyamangalam	10.9386°N	79.2375°E	Positive
14.	So 14	Someshwarapuram	10.9020°N	79.0989°E	Positive
15.	So 15	Thirupazhanam	10.8905°N	79.1343°E	Positive
16.	So 16	Sirkazhi	11.1616°N	79.7046°E	Positive
17.	So 17	Papanasam	10.9251°N	79.2708°E	Positive
18.	So 18	Puduchatiram	10.8528°N	78.9463°E	Positive
19.	So 19	Saliyantoppu	11.3672°N	79.6978°E	Positive
20.	So 20	Tanjore	10.8871°N	79.1230°E	Positive
21.	So 21	Vadakurangaduthurai	10.9230°N	79.1984°E	Positive
22.	So 22	Pudhur, S	11.2700°N	79.7213°E	Positive
23.	So 23	Ganapathiagaram	10.9061°N	79.1602°E	Positive
24.	So 24	Rayampettai,Tanjore	10.8871°N	79.1230°E	Positive
25.	So 25	Aduthurai	10.9932°N	79.4909°E	Positive
26.	So 26	Athukudi	11.1729°N	79.7078°E	Positive
27.	So 27	Sivapuri, Chidambaram	11.3838°N	79.7092°E	Positive
28.	So 28	Kallanai, Tanjore	10.8554°N	78.9412°E	Positive
29.	So 29	Govindhanallucheri	10.9374°N	79.2237°E	Positive
30.	So 30	Thiruvidaimaruthur	10.9979°N	79.4572°E	Positive
31.	So 31	Chellapatty, Madurai	9.94700°N	77.8899°E	Positive
32.	So 32	Bhavanisagar	11.4739°N	77.1465°E	Positive
33.	So 33	Dindugal	10.4091°N	78.0410°E	Positive
34.	So 34	Kodikulam, Madurai	9.9569°N	78.1607°E	Positive
35.	So 35	Samayapuram	10.9076°N	78.7065°E	Positive
36.	So 36	Perambalur	11.2300°N	78.8799°E	Positive
37.	So 37	Palaiyur, Thiruvarur	10.6648°N	79.4507°E	Positive
38.	So 38	Purathakudi	10.9360°N	78.7763°E	Positive
39.	So 39	Aduthurai	11.0139°N	79.4810°E	Positive
40.	So 40	Perugamani, Trichy	10.8708°N	78.5943°E	Positive
41.	So 41	Kumaramangalam	10.7403°N	78.6804°E	Positive
42.	So 42	Perugamani	10.8854°N	78.5414°E	Positive
43.	So 43	Orayur	10.8378°N	78.6483°E	Positive
44.	So 44	Vayalur	10.8240°N	78.6230°E	Positive
45.	So 45	Yethapur, Salem	11.5862°N	78.6992°E	Positive
46.	So 46	K. Sathanoor	10.8360°N	78.6591°E	Positive
47.	So 47	Maruthandakurichi	10.8372°N	78.6480°E	Positive
48.	So 48	Mochakottapalayam	10.9643°N	78.0242°E	Positive
49.	So 49	Melur, Madurai	10.0313°N	78.3382°E	Positive
50.	So 50	Kulithalai	10.8389°N	78.6523°E	Positive
51.	Nuclease free water	-	–	–	Negative

### 2.8 Validation of LAMP assay with naturally and artificially infected samples

The LAMP assay was validated with field samples using naturally infected rice seeds and artificial pathogen inoculation in seeds. For naturally infected seeds, seeds from the infected panicle were collected from rice fields in the districts of Coimbatore, Trichy and Villupuram. Three samples were collected from each district, and apparently healthy seeds collected from the panicle showing no infection were kept as a control. Contrarily, for artificial inoculation, seeds were surface sterilized with a 3% sodium hypochlorite solution for 5 minutes, followed by three washes with sterile water. *S. oryzae* spore suspension was added to rice seeds 24 hours before DNA extraction and dried overnight to remove the excess spore suspension on seed surface. Using the CTAB method, genomic DNA was extracted from rice seeds. According to Liang et al., 2016, 2 mg of rice seeds were ground in a mortar and pestle with 150 µL of extraction buffer (200 mmol/L Tris-Cl (pH 8.0), 50 mol/L ethylenediaminetetraacetic acid (EDTA; pH 8.0), 2.8 mol/L NaCl, 4% CTAB, and 2% sodium dodecyl sulphate). The obtained genomic DNA sample was digested with 10 µg/mL of RNase to remove any RNA contamination and stored at -20°C. The 2 µL final solution was directly used as a DNA template for the LAMP assay.

### 2.9 Validation of LAMP assay using weed species and different rice seed varieties

Following the procedure mentioned in section 2.3, template DNA was extracted from the weeds randomly collected from bunds of sheath rot infected fields. The LAMP assay was carried out using templates following the previously stated optimal conditions. When testing rice seeds of different varieties, 2 µl of total genomic DNA was utilized for the assay while maintaining all other parameters unchanged.

## 3 Results

### 3.1 PCR assay

In a PCR assay, fifty isolates of *S. oryzae* collected from diverse locations were amplified using the ITS 1 and 4 primers. The sequenced products were evaluated against other *S. oryzae* sequences found in the NCBI database. A similarity search was done in NCBI using the BLAST tool, the nucleotide sequence of the study isolate’s ITS region revealed 100% identity with other *S. oryzae* isolates and significantly very less similarity with other *Sarocladium* isolates.

### 3.2 Primer design

Using the Primer explorer V5 software programme, at least five different primer sets were predicted for unique sequences and used to build LAMP assay specific for *S. oryzae*. The primers were designed to allow *S. oryzae*-specific amplification showed 100% identity with 100% query coverage for *S. oryzae*. The G values of the 3′ end were determined during the design of LAMP primers and were less than -4 Kcal/mole. One pair of primers with a stable DNA construct and no primer-dimer formation were seen from *in silico* evaluation of the five designed primer sets generated by the software. Eventually, a set of six primers with high species specificity and sensitivity that targeted the *S. oryzae* β-tubulin sequences were elected for future investigation. The potential for the development of very weak to moderate secondary structures were found for the outer primers (forward-F3 and reverse-B3), moderate to weak secondary structures were identified for inner primers (FIP and BIP) whereas no to strong secondary structures were identified for loop primers (LF and LB) (**Supplementary Table 2**). The 3’ end of the primers was clamped with 2 to 3 guanine or/and cytosine bases (**Table 1**).

*In silico* hybridization of the primers revealed that the following six primers annealed to unique locations in 713 bp of the specific β -tubulin target sequence: F3 (444-463 bp), F2 (465-483 bp), LF (484-505 bp), F1c (517-537 bp), B1c (553-574 bp), LB (576-594 bp), B2 (600-618 bp) and B3 (624-643 bp). In **Figure 2**, binding positions of the primers were highlighted.

### 3.3 Optimization of LAMP assay reaction components and conditions

The critical components that could affect the effectiveness of the detection include the concentrations of MgSO_4_, betaine, *Bst* polymerase, dNTPs, and HNB dye. Magnesium ions in particular have a significant impact on the activity of DNA polymerase and primer annealing (Saiki et al., 1988; Yeh et al., 2005). Reuter et al. (2020) reported that the colour shift in the LAMP assay is due to the chelation of Mg^2 +^ ions by dNTPs. During the LAMP assay, the development of sky-blue colour signifies the positive reaction in the suspect samples (**Supplementary Figure 1**), whereas the violet colour signifies the negative reaction. The LAMP response was strongly impacted by the MgSO_4_ concentration. Colour change was established as the major benchmark for the optimization of MgSO_4_ concentration. The MgSO_4_ at 8 mM Conc. exhibited a deep sky blue colour in the LAMP reaction. The intensity of the sky blue colour decreased with either an increase or decrease from the optimal MgSO_4_ concentration. The intensity of sky blue decreased at concentrations of 4 mM, 6 mM, and 10 mM. Furthermore, the LAMP assay failed to produce colour at 0 mM and 2 mM concentration, indicating that MgSO_4_ is highly imperative for the LAMP assay. The results were validated using 2% agarose gel electrophoresis. In the presence of 8 mM MgSO_4_, a distinctive ladder-like pattern was visible; however, no such ladder-like pattern was found in the samples containing 0 mM, 2 mM, and 10 mM MgSO_4_ (**Supplementary Figure 1A**). Similarly, betaine at 0.8 mM concentration generated a characteristic ladder-like pattern, but other concentrations did not, implying negative results (**Supplementary Figure 1B**). The LAMP assay results at varying concentrations of *Bst* DNA polymerase (0U, 2U, 4U, 6U, and 8U) implied amplification at all doses except at 0 U. The best amplification, however, was found at a concentration of 2U *Bst* polymerase (**Supplementary Figure 1C**). The amplification improved noticeably when the dNTPs dose increased from 0.6 mM to 1.4 mM, as evidenced by brighter bands on 2% agarose gels (**Supplementary Figure 1D**). Hydroxy naphthol blue (HNB), an indicator dye for LAMP-based detection was utilized for the LAMP assay to enable visualization in the current work. According to the result of a range of HNB dye concentration test, concentration less than 120 µM have an impact on the visual detection of LAMP results but have no impact on the ladder-like pattern on 2% agarose gel. However, at greater concentrations affected the visualization as well as the ladder-like pattern (**Supplementary Figure 1E**).

A short range of temperatures (56°C to 70°C) (**Supplementary Figure 2A**) and minimum time (10-150 min) (**Supplementary Figure 2B**) were used to accurately assess the colour intensity using template DNA from the *S. oryzae* to find the optimum temperature and reaction time for the assay. The assay performed well at various temperatures. The outcomes were also evaluated on a 2% agarose gel. Positive results were seen after 30 min of incubation; however, no amplification was seen during the incubation period of 10 or 20 min. In case of different temperature range tested, positive results were obtained in all the cases but with varying range of colorimetric reaction and degree of amplification. However, conspicuous amplification and colorimetric reaction was seen at a temperature of 65°C. Therefore, *S. oryzae* was best detected at 65°C for 30 minutes under optimal circumstances.

### 3.4 Sensitivity of the LAMP assay

After establishing that the primer targeting the β-Tubulin gene became unique for *S. oryzae*, the lowest detection limit was interpreted with the use of 10-fold serial dilutions of *S. oryzae* DNA (100 ng to 1 fg). The least possible detection limit for *S. oryzae* as per reaction within 30 min incubation was 10 fg and was confirmed by colouration shift from violet to sky blue and reconfirmed by way of characteristic ladder-like pattern on 2% gel electrophoresis (**Figure 3**; **Table 3**). This experiment was repeated thrice.

### 3.5 Specificity of the LAMP assay

*S. oryzae* isolate and non-target DNA samples from 22 different phytopathogens listed in **Table 5** were used to test the primers’ specificity. The LAMP assay successfully identified *S. oryzae*, as shown by a colour change from violet to sky blue in the reaction solution, and did not cross-react with non-target phytopathogens, which retained their original violet colour. The specificity of the *S. oryzae* LAMP assay was validated by electrophoresis on 2% agarose gels stained with ethidium bromide. A characteristic ladder-like pattern was observed in reactions with genomic DNA from *S. oryzae* but not with DNA from other non-target phytopathogens. The findings demonstrated the great specificity of the newly designed LAMP primer targeting the β-Tubulin gene in identifying *S. oryzae* (**Figure 4**; **Table 4**).

### 3.6 Validation of LAMP assay

Primarily based on the colour development, the LAMP assay was initially optimized for *S. oryzae* pathogen to confirm the facilitative evaluation of a reaction with different isolates collected from different locations of Tamil Nadu. And it was reconfirmed by subjecting the samples to 2% agarose gel electrophoresis. A deep sky blue colour was visualized from the tubes retaining template DNA and HNB indicator dye. Meanwhile the violet colour indicated the absence of *S. oryzae* in the tubes with nuclease-free water (**Supplementary Figure 3**).

**Figure 5:**
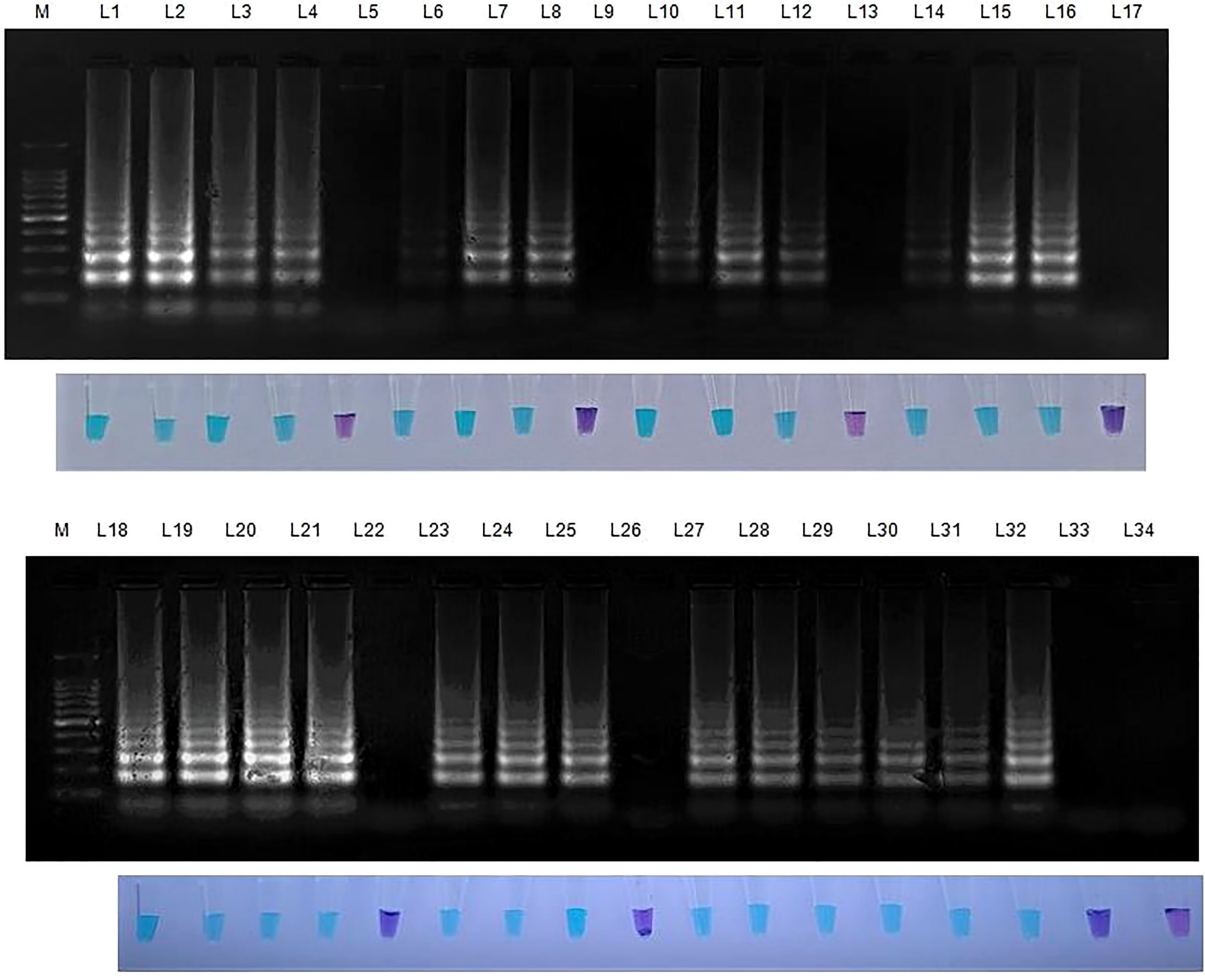
Validation of LAMP assay with *S. oryzae* infected field samples. Bright sky blue colour represents positive reaction while the violet colour indicates the negative reaction. Upper panel in the figure shows agarose gel electrophoresis results whereas lower panel shows reaction tubes for colorimetric detection. M- 100bp Ladder; Lane 1: *Sarocladium oryzae* isolate OM841519 (Positive control); Lane 2: Coimbatore infected sheath sample 1; Lane 3: Coimbatore infected sheath sample 2; Lane 4: Coimbatore infected sheath sample 3; Lane 5: Coimbatore healthy sheath sample; Lane 6: Coimbatore infected seed sample 1; Lane 7: Coimbatore infected seed sample 2; Lane 8: Coimbatore infected seed sample 3; Lane 9: Coimbatore healthy seed sample; Lane 10: Villupuram infected sheath sample 1; Lane11: Villupuram infected sheath sample 2; Lane 12: Villupuram infected sheath sample 3; Lane 13: Villupuram healthy sheath sample; Lane 14: Villupuram infected seed sample 1; Lane 15: Villupuram infected seed sample 2; Lane 16: Villupuram infected seed sample 3; Lane 17: Villupuram healthy seed sample; Lane 18: *Sarocladium oryzae* isolate OM841519 (Positive control); Lane 19: Trichy infected sheath sample 1; Lane 20: Trichy infected sheath sample 2; Lane 21: Trichy infected sheath sample 3; Lane 22: Trichy healthy sheath sample; Lane 23: Trichy infected seed sample 1; Lane 24: Trichy infected seed sample 2; Lane 25: Trichy infected seed sample 3; Lane 26: Trichy healthy seed sample; Lane 27: *Sarocladium oryzae* isolate OM841519 (Positive control); Lane 28: Artificially inoculated seed sample 1; Lane 29: Artificially inoculated seed sample 2; Lane 30: Artificially inoculated seed sample 3; Lane 31: Artificially inoculated seed sample 4; Lane 32: Artificially inoculated seed sample 5; Lane 33: Surface sterilized uninoculated seed sample; Lane 34: Nuclease free water (Negative Control).

The initial validation of the LAMP assay technique was performed on the *S. oryzae* culture sample. 30 samples were collected from several farmers’ fields and seed godowns for implementation at the field level. The LAMP assay was validated for field samples in each location using uninfected sample as a healthy control. The seeds from the infected panicle and the seeds with discolouration showed a positive reaction (deep sky blue colour), whereas the samples without infection showed a negative reaction (violet colour). In case of artificially inoculated seed samples, those seeds which are inoculated with pathogen showed colour development whereas, those which are surface sterilized didn’t developed colour resulting in negative reaction. Similar negative results were seen in nuclease-free water, which served as the negative control (**Figure 5**).

**Figure 6:**
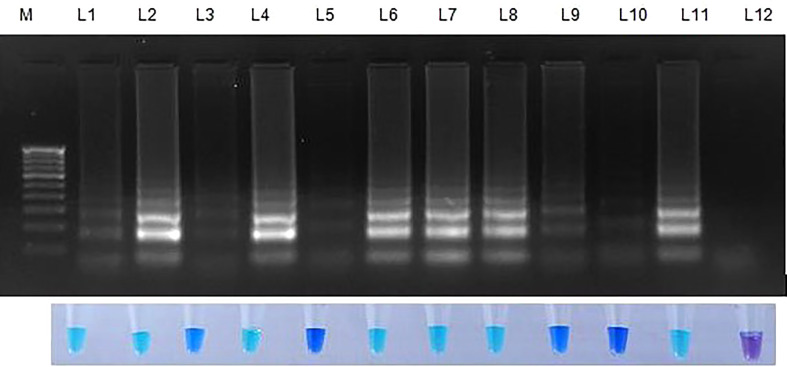
Validation of LAMP assay with *S. oryzae* infected paddy and weed samples. Bright sky blue colour represents positive reaction while the violet colour indicates the negative reaction. Total DNA isolated from fungi was used as the positive control. Upper panel in the figure shows agarose gel electrophoresis results whereas lower panel shows reaction tubes for colorimetric detection. M- 100bp Ladder; Lane 1: *Oryza sativa*; Lane 2: *Echinchloa crusgalli*: Lane 3: *Cyperus difformis*; Lane 4: *Eleusine indica*; Lane 5: *Dactyloctenium aegyptium*; Lane 6: *Cyperus iris*; Lane 7: *Eragrostis amabilis*; Lane 8: *Leersia hexandra*; Lane 9: *Phalaris minor*; Lane 10: *Brachiaria mutica*; Lane 11: *Sarocladium oryzae* isolate OM841519 (Positive control); Lane 12: Nuclease free water.

When weed samples were evaluated utilizing Tub LAMP primer sets, all weed species and rice sheath rot infected samples generated positive outcomes. However, the degree of amplification varied among the weed species. No amplification was observed in NTC (**Figure 6** and **Table 6**).

**Table 6:** Validation of LAMP with weeds collected from paddy fields.

S. No.	Weeds	Results
1.	*Echinchloa crusgalli*	**+**
2.	*Cyperus difformis*	**+**
3.	*Eleusine indica*	**+**
4.	*Dactyloctenium aegyptium*	**+**
5.	*Cyperus iris*	**+**
6.	*Eragrostis amabilis*	**+**
7.	*Leersia hexandra*	**+**
8.	*Phalaris minor*	**+**
9.	*Brachiaria mutica*	**+**
10.	Nuclease free water	**-**

**Figure 7:**
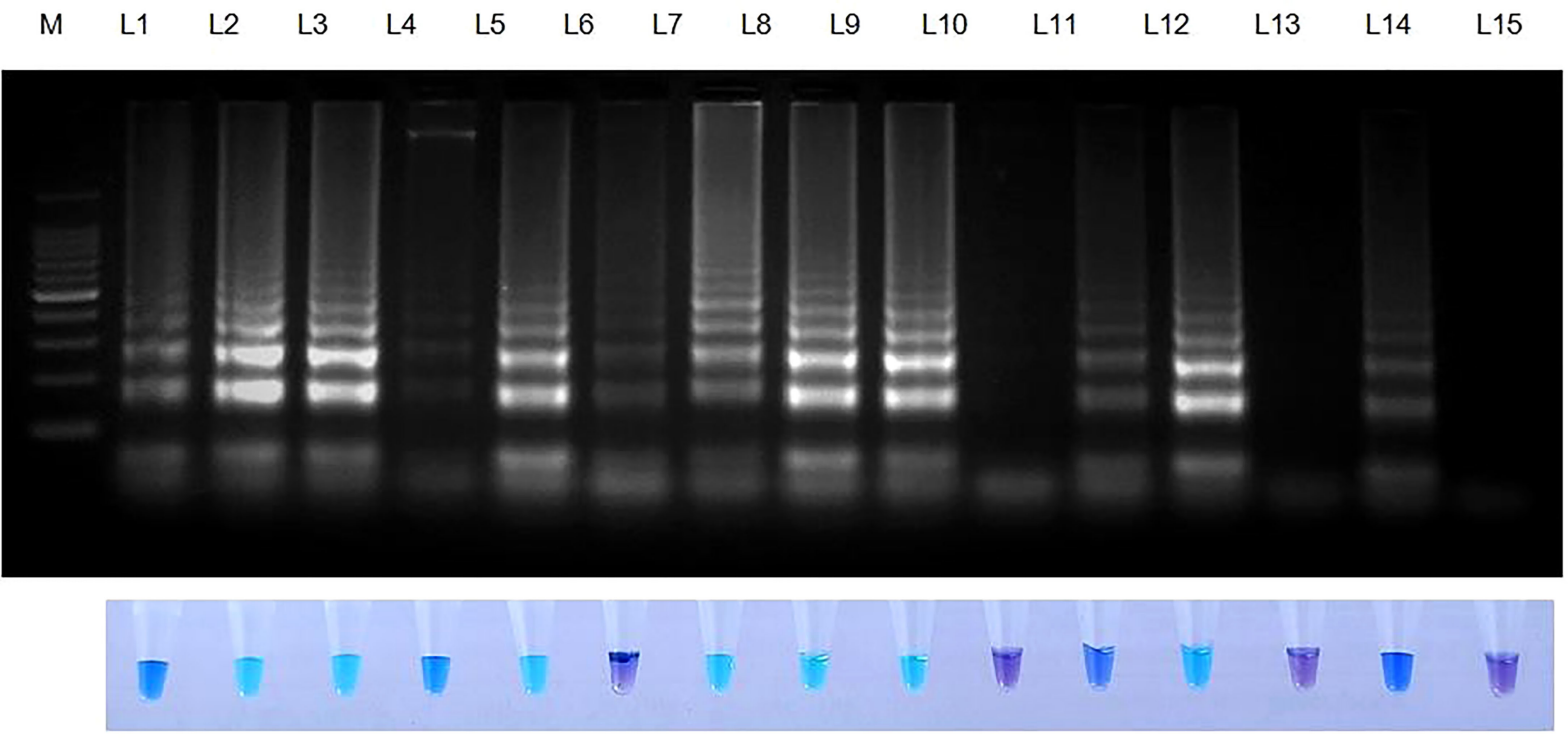
Validation of LAMP assay with naturally infected seeds of different varieties. Bright sky blue colour represents positive reaction while the violet colour indicates the negative reaction. Upper panel in the figure shows agarose gel electrophoresis results whereas lower panel shows reaction tubes for colorimetric detection. M- 100bp Ladder; Lane 1: *Sarocladium oryzae* isolate OM841519 (Positive control); Lane 2: Co 39; Lane 3: Co 51; Lane 4: Co 52; Lane 5: Co 53; Lane 6: Co 54; Lane 7: Co 55; Lane 8: ADT 37; Lane 9: ADT 43; Lane 10: RNR 15048; Lane11: TN 1; Lane 12: TPS 5; Lane 13: TRY 43; Lane 14: ASD 16; Lane 15: Nuclease free water (Negative Control).

Rice seeds from 11 of 13 rice varieties have showed positive reaction for *S. oryzae* (**Table 7**). Two varieties namely, TRY 43 and RNR 15048 did not exhibit amplification (**Figure 7**) along with NTC.

**Table 7:** Validation of LAMP assay with different varieties of rice.

S. No.	Rice seed variety	Results
1.	Co 39	**+**
2.	Co 51	**+**
3.	Co 52	**+**
4.	Co 53	**+**
5.	Co 54	**+**
6.	Co 55	**+**
7.	ADT 37	**+**
8.	ADT 43	**+**
9.	RNR 15048	**-**
10.	TN 1	**+**
11.	TPS 5	**+**
12.	TRY 43	**-**
13.	ASD 16	**+**
14.	Nuclease free water	**-**

## 4 Discussion

Seeds are the prime carriers of numerous phytopathogens that cause most plant diseases, resulting in significant agricultural output loss (Neergaard, 1977). Notably, it is well admitted that both major and minor fungal pathogens of rice were spread by seeds. *S. oryzae* is one of the rice seed-borne infections that cause the catastrophic disease called sheath rot, which is widespread in the major rice-producing countries (Richardson, 1981; Reddy and Sathyanarayana, 2001; Dossou and Silue, 2018). To reduce the possibility of disease outbreaks, it is crucial to identify the seed-borne inoculum so that early control measures like seed treatment, seed certification and quarantine legislation may be implemented.

Presently, the International Seed Testing Association, 2017 (ISTA) proposes the technique of blotting a working sample of 400 seeds divided into 25 seed subsamples onto a 90 mm filter paper saturated with distilled water and documenting the proportion of diseased seed after incubating for 7 days at 27 ± 2°C in alternate 12 hr. cycles of light and darkness followed by validation with stereoscopic observation of each seed. Despite the simplicity of the blotting procedure and its widespread usage in diagnostic labs, a proper inspection calls for skilled diagnosticians who can spot the conidia of the ensuing fungal growth *via* a stereomicroscope. One factor to consider during this process is the prevalence of other conidia with indistinguishable morphological traits as in saprophytic fungus *Verticillium* spp. that might be misdiagnosed as *S. oryzae*. Any enterprise seeking to certify the absence of pathogens in rice seed faces economic losses as a result of misdiagnosis. In addition, blotting requires a lengthy incubation period of seven days, which is another drawback of this technique. The tests also necessitate the use of vast, high-priced and controlled-environment chambers.

To tackle this challenge, quick and reliable identification of *S. oryzae* is crucial for elucidating the pathogenic determinants and core mechanisms. Consequently, corresponding disease management strategies can be devised. Rapid and precise detection of an appropriately low amount of pathogen is crucial to diagnose *S. oryzae* at the seed for disease surveillance and management activities.

In well-equipped laboratories, PCR-based procedures are frequently employed to perform routine identification tests. However, these technologies are sophisticated and time-consuming, as it decreases the likelihood of on-site sampling and detection, prolonging the period between sampling and results (Boonham et al., 2008). Also, PCR-based approach has a few limitations, one of which is its vulnerability to inhibitors, which might affect the specificity and potentially yield false negative results. These inhibitors include a diverse range of compounds like phenols, polysaccharides, melanin, humic or tannic acids, and rice seed proteins that have received little research (Tian et al., 2004; Schrader et al., 2012). The LAMP test has excellent characteristics since it provides for quick, sensitive, specific, and simplistic field detection and is perhaps less susceptible to inhibitors (Kaneko et al., 2007).

Another prime flaw of PCR is that it only detects two amplicons in the target gene; therefore there is a higher likelihood of false-positive reactions occurring. Whereas, LAMP uses a set of six primers to amplify six different target regions, it is highly specific and has a reduced chance of false-positive results than traditional PCR (Marimuthu et al., 2020).

LAMP technique is strongly recommended as a fast diagnostic method since it may amplify the DNA in less than 60 minutes, which is significantly faster than traditional PCR (Notomi et al., 2000). Since LAMP amplifies DNA in an isothermal condition, it may be completed in a single reaction under visual examination. Magnesium pyrophosphate being deposited during the reaction period results in an increase in the turbidity of the amplified products (a positive reaction), which in turn cuts down on the amount of time needed for reconfirmation under gel electrophoresis (Fukuta et al., 2004).

The majority of formerly described LAMP assays for fungi target areas with significant interspecies similarity, like the ITS (Zeng et al., 2017), which tends to have less interspecific variability and may impede the synthesis of species-specific primers. LAMP primers were developed using the sequence of the conserved β-tubulin gene to achieve excellent *S. oryzae* specificity. The primers developed for this study were more precise and sensitive in distinguishing *Sarocladium oryzae* from other fungal phytopathogens. The developed LAMP primers were capable of detecting *S. oryzae* DNA at inoculum levels as low as 10 fg. This reveals that this assay is 100 times more sensitive than traditional PCR. Because of its extreme sensitivity, LAMP was an exemplary technique for diagnosing *S. oryzae* even at minimal concentrations.

Prasannakumar et al., 2021 reported *S. oryzae* identification using RNA polymerase II largest subunit region-based LAMP analysis. In this technique, the LAMP methodology was used for onsite detection of *S. oryzae* seed-borne inoculum through the construction of a LAMP based foldable device, and it was shown that the LAMP assay was more sensitive and has a detection limit of up to 50 fg of genomic DNA when compared to traditional PCR (100 pg). They investigated the specificity of the LAMP assay with six other phytopathogens and concluded that RNA polymerase II largest subunit-based LAMP primer solely unique to *S. oryzae*. Moreover, our findings also exhibit a significant correlation with Ghosh et al. (2015) and Lakshmi et al. (2022), who devised the LAMP assay for the detection of *Fusarium oxysporum* f. sp. *ciceris*, the causative agent of chickpea wilt and *Bipolaris oryzae*, the causal organism of brown spot of rice.

The LAMP test was performed at 65°C for 30 minutes and did not require the use of a programmed thermocycler. Before amplification, the LAMP was optimized by adding a visualization indicator, hydroxy naphthol blue (HNB) dye. This was done to avoid the use of intercalating dyes like SYBR green (Parida et al., 2005), which can exacerbate the frequencies of aerosol contamination when the tubes were opened to add. Most of the non-specific detection and misleading false positive/negative result that occurs during LAMP reaction was mainly due to cross-contamination that may arise because of the possibility of cis and trans priming of primer (Zaczek-Moczydłowska et al., 2020). This has been circumvented by the effective use of metal ion indicators such as calcein and HNB dye, to visualize the final result of the LAMP reaction (Tomita et al., 2008; Wang et al., 2012). The results of the assay were indicated by the colour shift when this metal ion indicator was added to the pre-reaction solution. In the presence of magnesium ions, the HNB metal indicator changes colour from violet to sky blue (Goto et al., 2009). The LAMP reaction results in the depletion of magnesium ions in the solution which causes a colour shift from violet to blue, signifying a positive reaction. This colour change was due to the interaction of magnesium ions with pyrophosphate produced by dNTPs during the LAMP reaction resulting in a precipitate (Marimuthu et al., 2020; Reuter et al., 2020). The detection limit of the HNB-LAMP test was reported to be analogous to that of the assay employing SYBR green dye by Goto et al. (2009) while comparing the LAMP assay using HNB, SYBR green and calcein dyes. Contrarily, Balne et al. (2013) reported the detection threshold of the LAMP test containing calcein was 10 times lower than that of other assays.

The sensitivity of the LAMP test was confirmed for DNA samples at different concentration. Also, LAMP precisely recognized only *S. oryzae* DNA, and no cross response was observed in other phytopathogens used in this study. Owing to this, only the LAMP test will allow for the early and accurate detection of seed-borne *S. oryzae*.

To validate the LAMP assay, naturally infected and uninfected field samples collected from three distinct villages were screened for *S. oryzae* detection. All the collected samples were tested using LAMP assay, and all infected samples showed positive result when compared to the healthy control, which indicates the detection efficiency of *S. oryzae* in a short period. In addition, weeds around the sheath rot-infected rice field and seeds of several rice kinds were tested for *S. oryzae* detection for final validation.

As the LAMP technique is remarkably simplistic, efficient and incredibly specific, it is ideal for the development of diagnostic tools for accurate and timely detection of *S. oryzae* in seeds as well as for the forecasting of disease outbreaks. This assay will aid in the successful management of rice sheath rot by detecting the *S. oryzae* infected seeds in quarantine station and seed testing laboratories and implementing prophylactic steps in the field.

## 5 Conclusion

It is concluded that the LAMP technique has a very high specificity and sensitivity for the detection of *S. oryzae*. The LAMP approach eliminates the requirement for sophisticated equipment such as PCR machines, gel electrophoresis, or gel imaging systems for detection and visualization. Finally, the LAMP primers are specific for the detection of *S. oryzae*. This assay has the potential to be a future on-site diagnostic tool since it is fast, sensitive, needs less equipment, and requires less specialized labour. Our findings will contribute to the body of knowledge on reliable approaches for the early detection of *S. oryzae* from seeds, facilitating the adoption of appropriate prophylactic measures, maintaining high-quality seed standards, and preventing catastrophic field epidemics.

## Data availability statement

The datasets presented in this study can be found in online repositories. The names of the repository/repositories and accession number(s) can be found in the article/**Supplementary Material**.

## Author contributions

CG devised and planned the experiments. RL carried out the experiments, collected the data, and wrote the initial draft of the manuscript. The manuscript’s initial draft was revised by CG. RL updated and refined the draft. All authors contributed to the article and approved the submitted version.

## Acknowledgments

We gratefully acknowledge the DST-FIST Lab, Tamil Nadu Agricultural University, Coimbatore, Tamil Nadu, India. Thanks to Dr. M. Jayakanthan of the Department of Plant Molecular Biology and Bioinformatics, Tamil Nadu Agricultural University, Coimbatore, India for his guidance on targeting gene and primer designing.

## Conflict of interest

The authors declare that the research was conducted in the absence of any commercial or financial relationships that could be construed as a potential conflict of interest.

## Publisher’s note

All claims expressed in this article are solely those of the authors and do not necessarily represent those of their affiliated organizations, or those of the publisher, the editors and the reviewers. Any product that may be evaluated in this article, or claim that may be made by its manufacturer, is not guaranteed or endorsed by the publisher.

## References

[B1] Abbade E. B. (2021). Estimating the potential for nutrition and energy production derived from maize (*Zea mays* l.) and rice (*Oryza sativa* l.) losses in Brazil. Waste Manage. 134, 170–176. doi: 10.1016/j.wasman.2021.08.009 34425385

[B2] Altschul S. F. Gish W. Miller W. Myers E. W. Lipman D. J. (1990). Basic local alignment search tool. J. Mol. Biol. 215, 403–410. doi: 10.1016/S0022-2836(05)80360-2 2231712

[B3] Ayyadurai N. Kirubakaran S. I. Srisha S. Sakthivel N. (2005). Biological and molecular variability of *Sarocladium oryzae*, the sheath rot pathogen of rice (*Oryza sativa* l.). Curr. Microbiol. 50, 319–323. doi: 10.1007/s00284-005-4509-6 15968500

[B4] Balne P. K. Barik M. R. Sharma S. Basu S. (2013). Development of a loop-mediated isothermal amplification assay targeting the mpb64 gene for diagnosis of intraocular tuberculosis. J. Clin. Microbiol. 51, 3839–3840. doi: 10.1128/JCM.01386-13 23966513 PMC3889776

[B5] Bigirimana V. P. Hua G. K. Nyamangyoku O. I. Höfte M. (2015). Rice sheath rot: An emerging ubiquitous destructive disease complex. Front. Plant Sci. 6. doi: 10.3389/fpls.2015.01066 PMC4675855 26697031

[B6] Boonham N. Glover R. Tomlinson J. Mumford R. (2008). “Exploiting generic platform technologies for the detection and identification of plant pathogens,” in Sustainable disease management in a European context (Dordrecht: Springer), 355–363.

[B7] Dossou B. Silue D. (2018). Rice pathogens intercepted on seeds originating from 11 African countries and from the USA. Seed Sci. Technol. 46, 31–40. doi: 10.15258/sst.2018.46.1.03

[B8] Doyle J. (1991). “DNA Protocols for plants,” in Molecular techniques in taxonomy (Berlin, Heidelberg: Springer), 283–293.

[B9] Fukuta S. Mizukami Y. Ishida A. Ueda J. Hasegawa M. Hayashi I.. (2004). Real-time loop-mediated isothermal amplification for the CaMV-35S promoter as a screening method for genetically modified organisms. Eur. Food Res. Technol. 218, 496–500. doi: 10.1007/s00217-003-0862-5

[B10] Ghosh R. Nagavardhini A. Sengupta A. Sharma M. (2015). Development of loop-mediated isothermal amplification (LAMP) assay for rapid detection of *Fusarium oxysporum* f. sp. *ciceris*-wilt pathogen of chickpea. BMC Res. Notes 8, 1–10. doi: 10.1186/s13104-015-0997-z 25886622 PMC4332723

[B11] Gopalakrishnan C. Kamalakannan A. Valluvaparidasan V. (2010). Effect of seed-borne *Sarocladium oryzae*, the incitant of rice sheath rot on rice seed quality. J. Plant Prot. Res. 50, 98–102. doi: 10.2478/v10045-010-0017-1

[B12] Goto M. Honda E. Ogura A. Nomoto A. Hanaki K. I. (2009). Colorimetric detection of loop-mediated isothermal amplification reaction by using hydroxy naphthol blue. Biotechniques 46, 167–172. doi: 10.2144/000113072 19317660

[B13] ISTA (International Seed Testing Association) (2017). “International rules for seed testing. proceedings of the international seed testing association,” in Bassersdorf (Switzerland: Seed Science and Technology).

[B14] Kaneko H. Kawana T. Fukushima E. Suzutani T. (2007). Tolerance of loop-mediated isothermal amplification to a culture medium and biological substances. J. Biochem. Biophys. Methods 70, 499–501. doi: 10.1016/j.jbbm.2006.08.008 17011631

[B15] Lakshmi K. R. Kamalakannan A. Gopalakrishnan C. Rajesh S. Panneerselvam S. Ganapati P. S. (2022). Loop-mediated isothermal amplification assay: A specific and sensitive tool for the detection of *Bipolaris oryzae* causing brown spot disease in rice. Phytoparasitica 50, 543–553. doi: 10.1007/s12600-022-00979-3

[B16] Liang H. Deng Y. Wang C. Xu X. (2016). A high-throughput DNA extraction method from rice seeds. Biotechnol. Biotechnol. Equip. 30, 32–35. doi: 10.1080/13102818.2015.1088401

[B17] Mancini V. Murolo S. Romanazzi G. (2016). Diagnostic methods for detecting fungal pathogens on vegetable seeds. Plant Pathol. 65, 691–703. doi: 10.1111/ppa.12515

[B18] Marimuthu K. Ayyanar K. Varagur Ganesan M. Vaikuntavasan P. Uthandi S. Mathiyazhagan K.. (2020). Loop-mediated isothermal amplification assay for the detection of plasmopara viticola infecting grapes. J. Phytopathol. 168, 144–155. doi: 10.1111/jph.12866

[B19] Mathur S. C. (1981). Observations on diseases of dryland rice in Brazil. Int. Rice Res. Newsl. 6, 11–12.

[B20] Mew T. W. Gonzales P. (2002). A handbook of rice seedborne fungi (Philippines: International Rice Research Institute).

[B21] Nasruddin A. Amin N. (2013). Effects of cultivar, planting period, and fungicide usage on rice blast infection levels and crop yield. J. Agric. Sci. 5, 160. doi: 10.5539/jas.v5n1p160

[B22] Neergaard P. (1977). “Economic significance of seed-borne diseases,” in The seed pathology (Palgrave, London: Red Globe Press), 3–39.

[B23] Notomi T. Okayama H. Masubuchi H. Yonekawa T. Watanabe K. Amino N.. (2000). Loop-mediated isothermal amplification of DNA. Nucleic Acids Res. 28, 63. doi: 10.1093/nar/28.12.e63 PMC102748 10871386

[B24] Parida M. Horioke K. Ishida H. Dash P. K. Saxena P. Jana A. M.. (2005). Rapid detection and differentiation of dengue virus serotypes by a real-time reverse transcription-loop-mediated isothermal amplification assay. J. Clin. Microbiol. 43, 2895–2903. doi: 10.1128/JCM.43.6.2895-2903.2005 15956414 PMC1151941

[B25] Pearce D. A. Bridge P. D. Hawksworth D. L. (2001). “Species concept in sarocladium, the causal agent of sheath rot in rice and bamboo blight,” in Major fungal diseases of rice (Dordrecht: Springer), 285–292.

[B26] Prasannakumar M. K. Parivallal P. B. Pramesh D. Mahesh H. B. Raj E. (2021). LAMP-based foldable microdevice platform for the rapid detection of *Magnaporthe oryzae* and *Sarocladium oryzae* in rice seed. Sci. Rep. 11, 1–10. doi: 10.1038/s41598-020-80644-z 33420312 PMC7794292

[B27] Reddy O. R. Sathyanarayana N. (2001). “Seed-borne fungi of rice and quarantine significance,” in Major fungal diseases of rice (Dordrecht: Springer), 331–345.

[B28] Reuter C. Slesiona N. Hentschel S. Aehlig O. Breitenstein A. Csáki A.. (2020). Loop-mediated amplification as promising on-site detection approach for *Legionella pneumophila* and legionella spp. App. Microbiol. Biotechnol. 104, 405–415. doi: 10.1007/s00253-019-10286-3 31832709

[B29] Richardson M. J. (1981). Supplement 1 to an annotated list of seed-borne diseases. 3rd ed (Zurich, Switzerland: The International Seed Testing Association).

[B30] Saiki R. K. Gelfand D. H. Stoffel S. Scharf S. J. Higuchi R. Horn G. T.. (1988). Primer-directed enzymatic amplification of DNA with a thermostable DNA polymerase. Science 239, 487–491. doi: 10.1126/science.2448875 2448875

[B31] Sarangi S. K. Islam M. R. (2019). “Advances in agronomic and related management options for sundarbans,” in The sundarbans: a disaster-prone eco-region (Cham: Springer), 225–260.

[B32] Schrader C. Schielke A. Ellerbroek L. Johne R. (2012). PCR inhibitors – occurrence, properties and removal. J. Appl. Microbiol. 113, 1014–1026. doi: 10.1111/j.1365-2672.2012.05384.x 22747964

[B33] Teng P. S. (1994). Integrated pest management in rice. Exp. Agric. 30, 115–137. doi: 10.1017/S001447970002408X

[B34] Tian C. Wan P. Sun S. Li J. Chen M. (2004). Genome-wide analysis of the GRAS gene family in rice and *Arabidopsis* . Plant Mol. Biol. 54, 519–532. doi: 10.1023/B:PLAN.0000038256.89809.57 15316287

[B35] Tiwari A. K. (2016). Current trends in plant disease diagnostics and management practices (Berlin: Springer), 207–219. doi: 10.1007/978-3-319-27312-9

[B36] Tomita N. Mori Y. Kanda H. Notomi T. (2008). Loop-mediated isothermal amplification (LAMP) of gene sequences and simple visual detection of products. Nat. Protoc. 3, 877–882. doi: 10.1038/nprot.2008.57 18451795

[B37] Wang X. Zhang Q. Zhang F. Ma F. Zheng W. Zhao Z.. (2012). Visual detection of the human metapneumovirus using reverse transcription loop-mediated isothermal amplification with hydroxynaphthol blue dye. Virol. J. 9, 1–6. doi: 10.1186/1743-422X-9-138 22838725 PMC3487928

[B38] Wastling S. L. Picozzi K. Kakembo A. S. L. Welburn S. C. (2000). LAMP for human African trypanosomiasis: a comparative study of detection formats. PloS Neglec. Trop. D. 4, 865. doi: 10.1371/journal.pntd.0000865 PMC2970543 21072228

[B39] Yeh H. Y. Shoemaker C. A. Klesius P. H. (2005). Evaluation of a loop-mediated isothermal amplification method for rapid detection of channel catfish *Ictalurus punctatus* important bacterial pathogen *Edwardsiella ictaluri* . J. Microbiol. Methods 63, 36–44. doi: 10.1016/j.mimet.2005.02.015 16157211

[B40] Zaczek-Moczydłowska M. A. Mohamed-Smith L. Toldrà A. Hooper C. Campàs M. Furones M. D.. (2020). A single-tube HNB-based loop-mediated isothermal amplification for the robust detection of the ostreid herpesvirus 1. Int. J. Mol. Sci. 21, 6605. doi: 10.3390/ijms21186605 32917059 PMC7555478

[B41] Zeng D. Ye W. Xu M. Lu C. Tian Q. Zheng X. (2017). Rapid diagnosis of soya bean root rot caused by *Fusarium culmorum* using a loop-mediated isothermal amplification assay. J. Phytopathol. 165, 249–256. doi: 10.1111/jph.12556

[B42] Zhang S. Y. Dai D. J. Wang H. D. Zhang C. Q. (2019). One-step loop-mediated isothermal amplification (LAMP) for the rapid and sensitive detection of *Fusarium fujikuroi* in bakanae disease through NRPS31, an important gene in the gibberellic acid bio-synthesis. Sci. Rep. 9, 1–9. doi: 10.1038/s41598-019-39874-z 30842486 PMC6403233

[B43] Zhang X. Harrington T. C. Batzer J. C. Kubota R. Peres N. A. Gleason M. L. (2016). Detection of *Colletotrichum acutatum* sensu lato on strawberry by loop-mediated isothermal amplification. Plant Dis. 100, 1804–1812. doi: 10.1094/PDIS-09-15-1013-RE 30682979

