# Supplementary material for: A colorimetric hydroxy naphthol blue based loop-mediated isothermal amplification detection assay targeting the β-tubulin locus of Sarocladium oryzae infecting rice seed
Source: Front Plant Sci. 2022 Nov 21;13:1077328. doi: 10.3389/fpls.2022.1077328 (PMC9720317; doi:10.3389/fpls.2022.1077328)
Supplement: Supplementary file 1 [file DataSheet_1.doc]

Supplementary Material

| A. 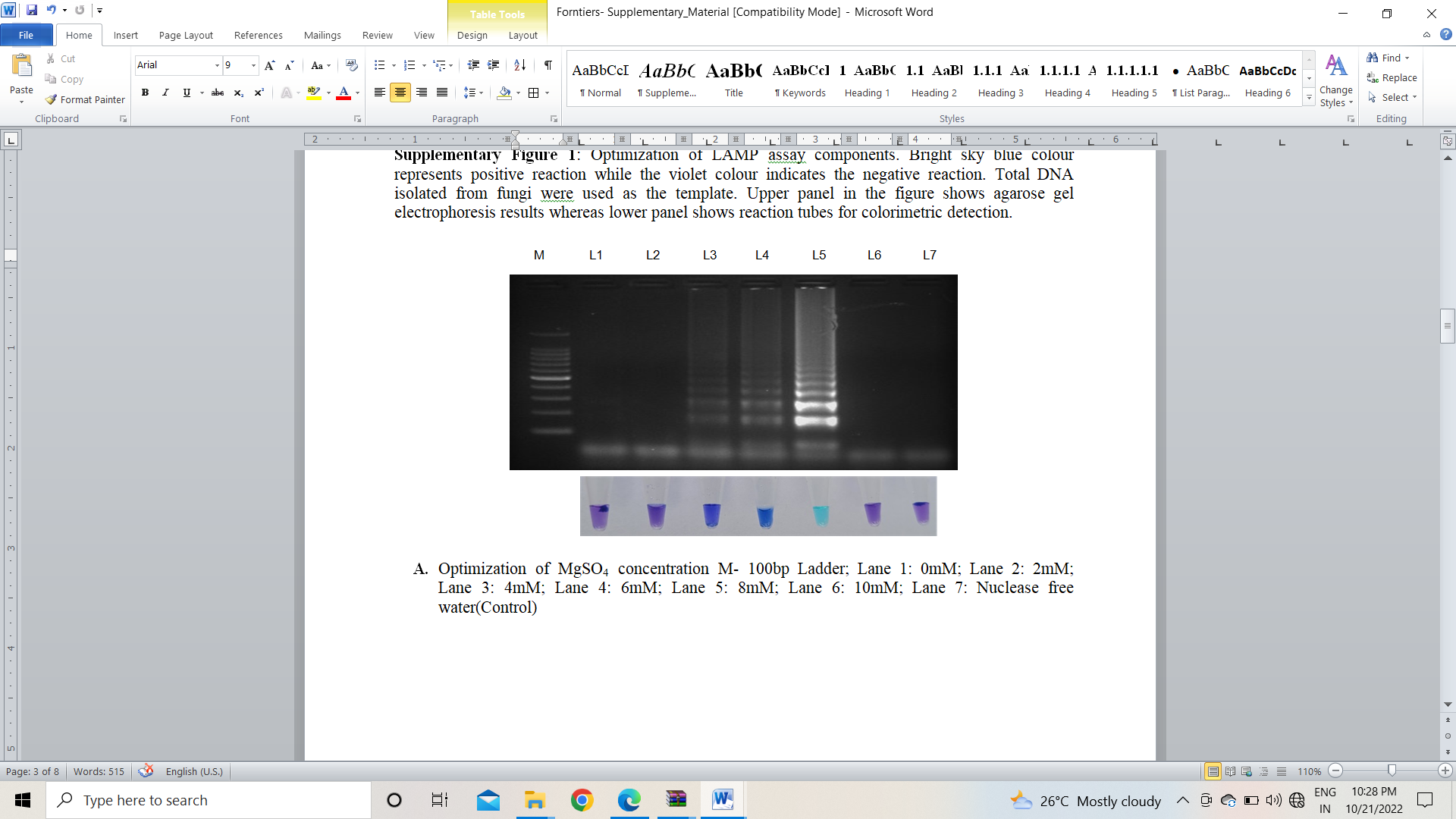 | B.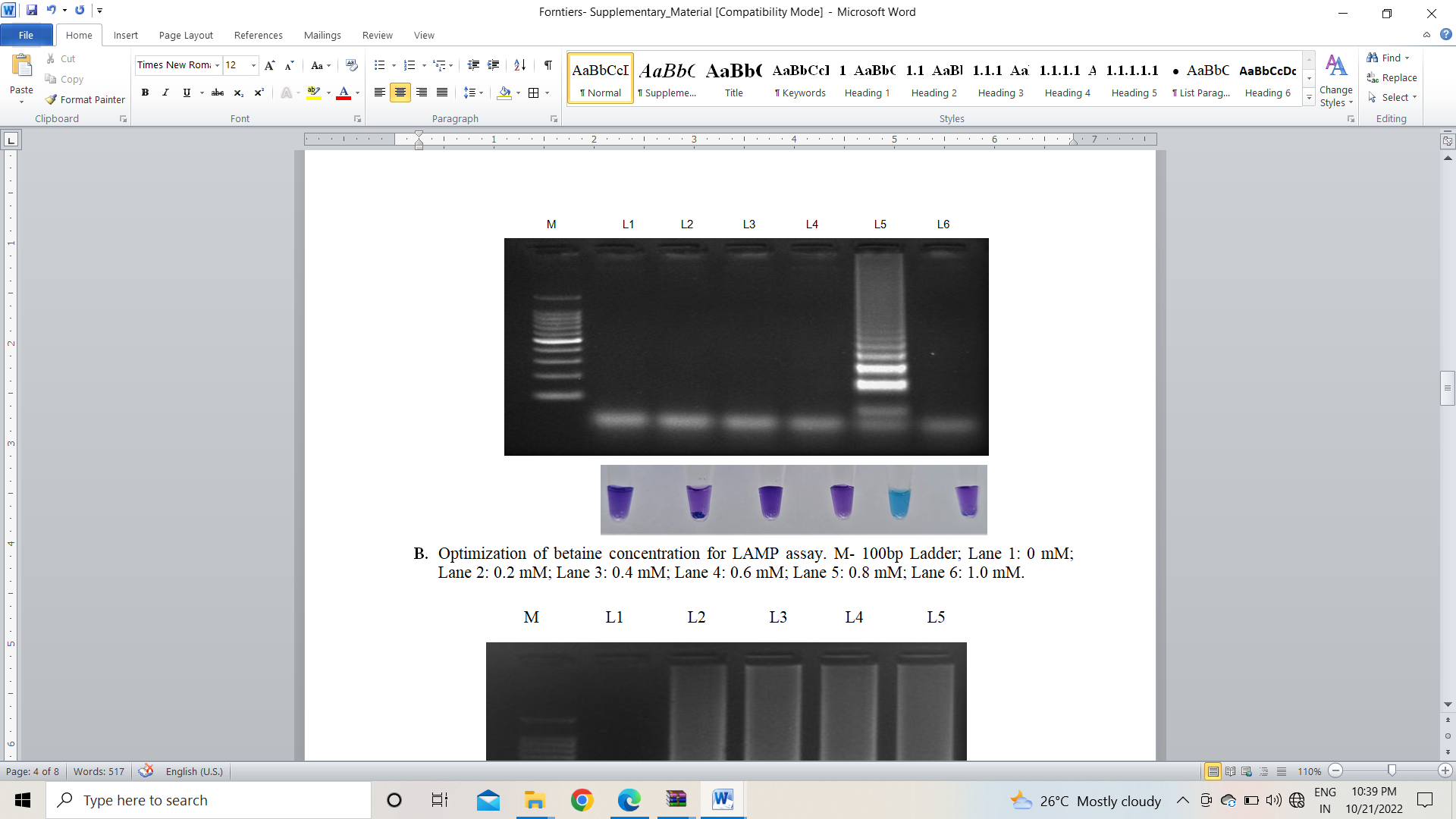 |
| --- | --- |
| C.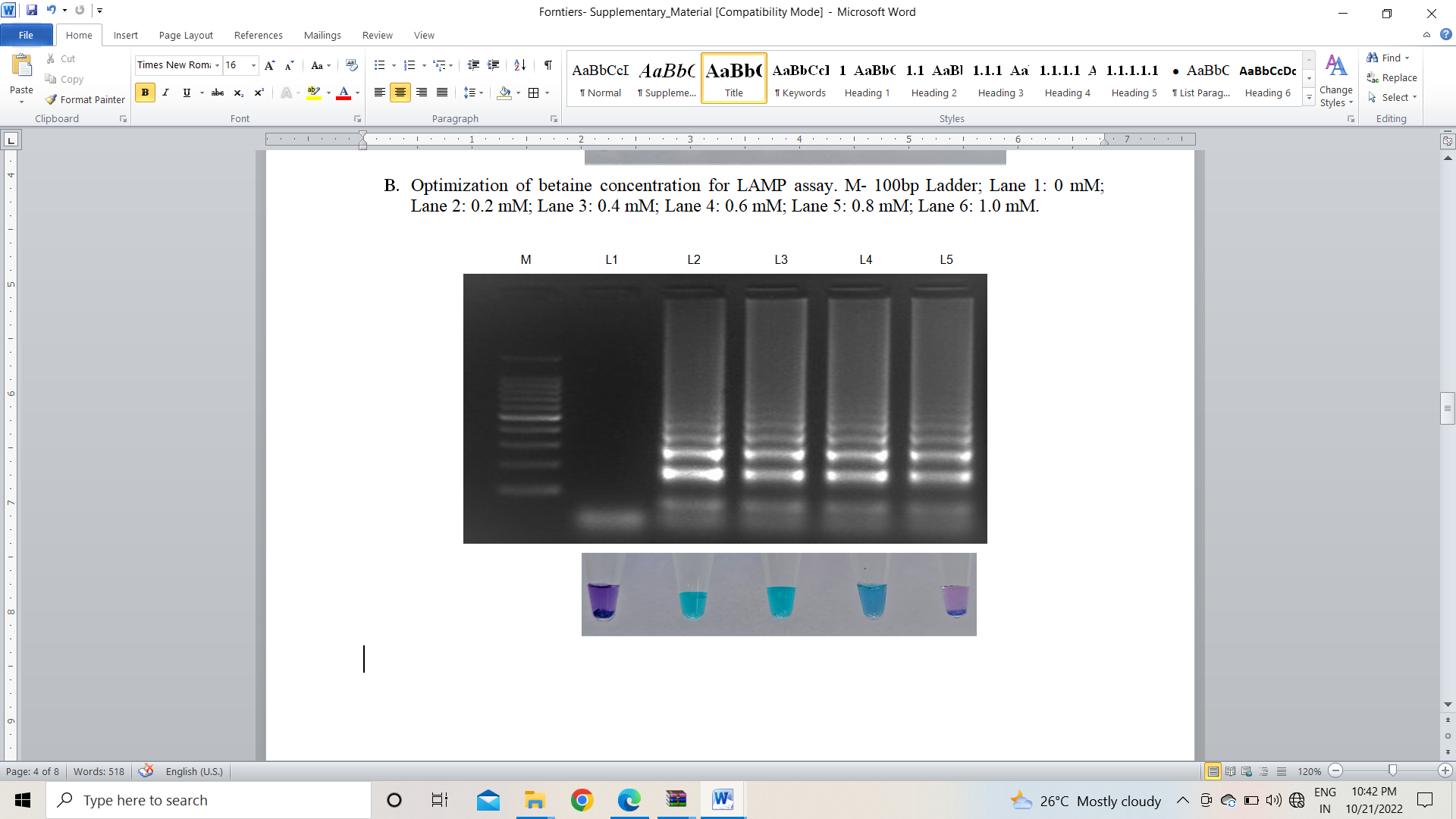 | D.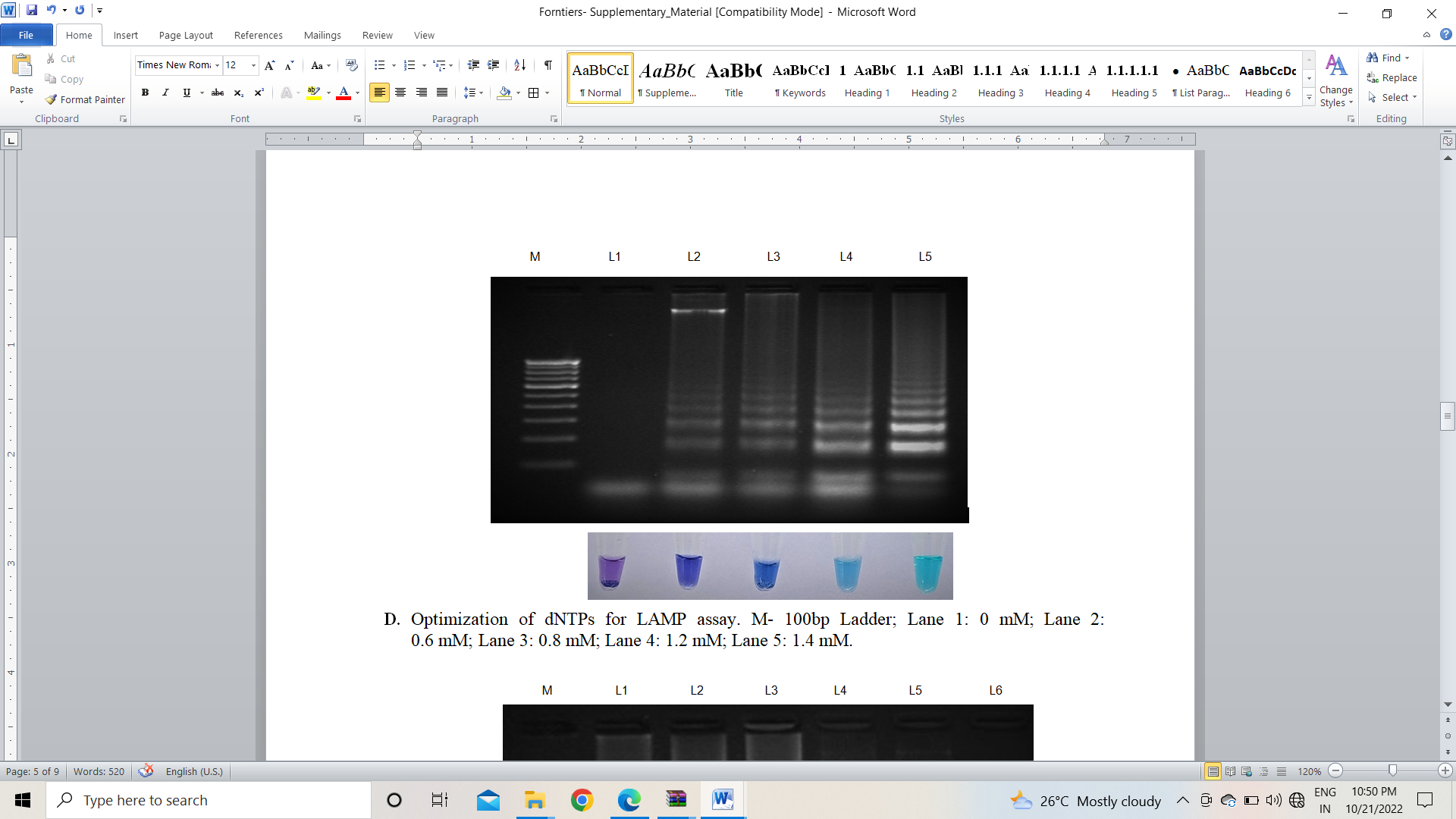 |
| E.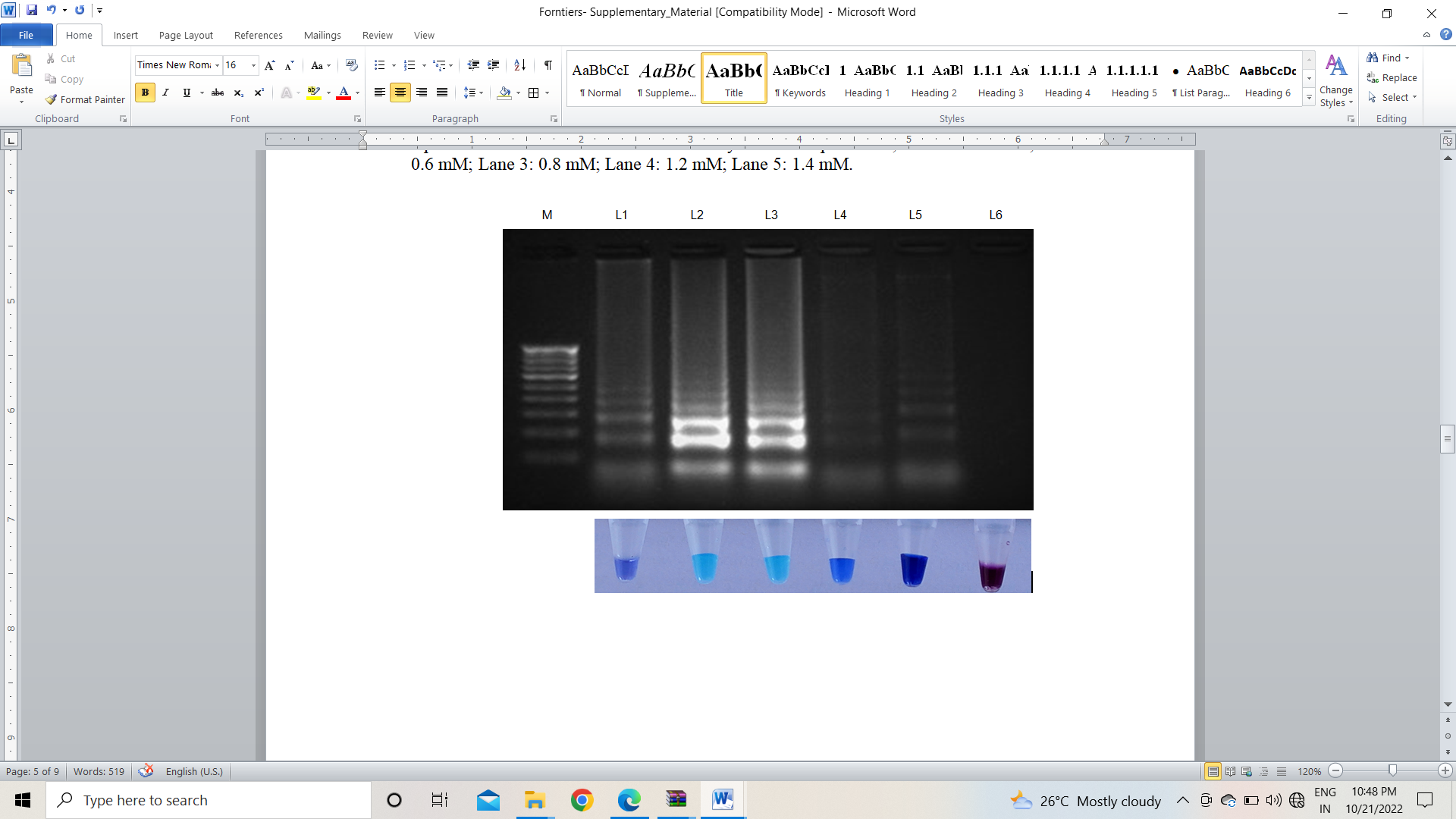 |  |

**Figure S1**: Optimization of LAMP assay components. Bright sky blue colour represents positive reaction while the violet colour indicates the negative reaction. Total DNA isolated from fungi were used as the template. Upper panel in the figure shows agarose gel electrophoresis results whereas lower panel shows reaction tubes for colorimetric detection. M- 100bp Ladder **A.** MgSO_4_ concentration Lane 1-7: 0mM, 2mM, 4mM, 6mM, 8mM, 10mM, Nuclease free water(Control). **B. B**etaine concentration Lane 1-6: 0 mM, 0.2 mM, 0.4 mM, 0.6 mM, 0.8 mM, 1.0 mM. **C.** *Bst* DNA Polymerase Lane 1-5: 0U, 2U, 4U, 6U, 8U **D.** dNTPs concentration Lane 1-5: 0 mM, 0.6 mM, 0.8 mM, 1.2 mM, 1.4 mM. **E.** HNB dyes Lane 1-5: 80 µM, 100 µM, 120 µM, 148 µM, 300 µM.

| A. 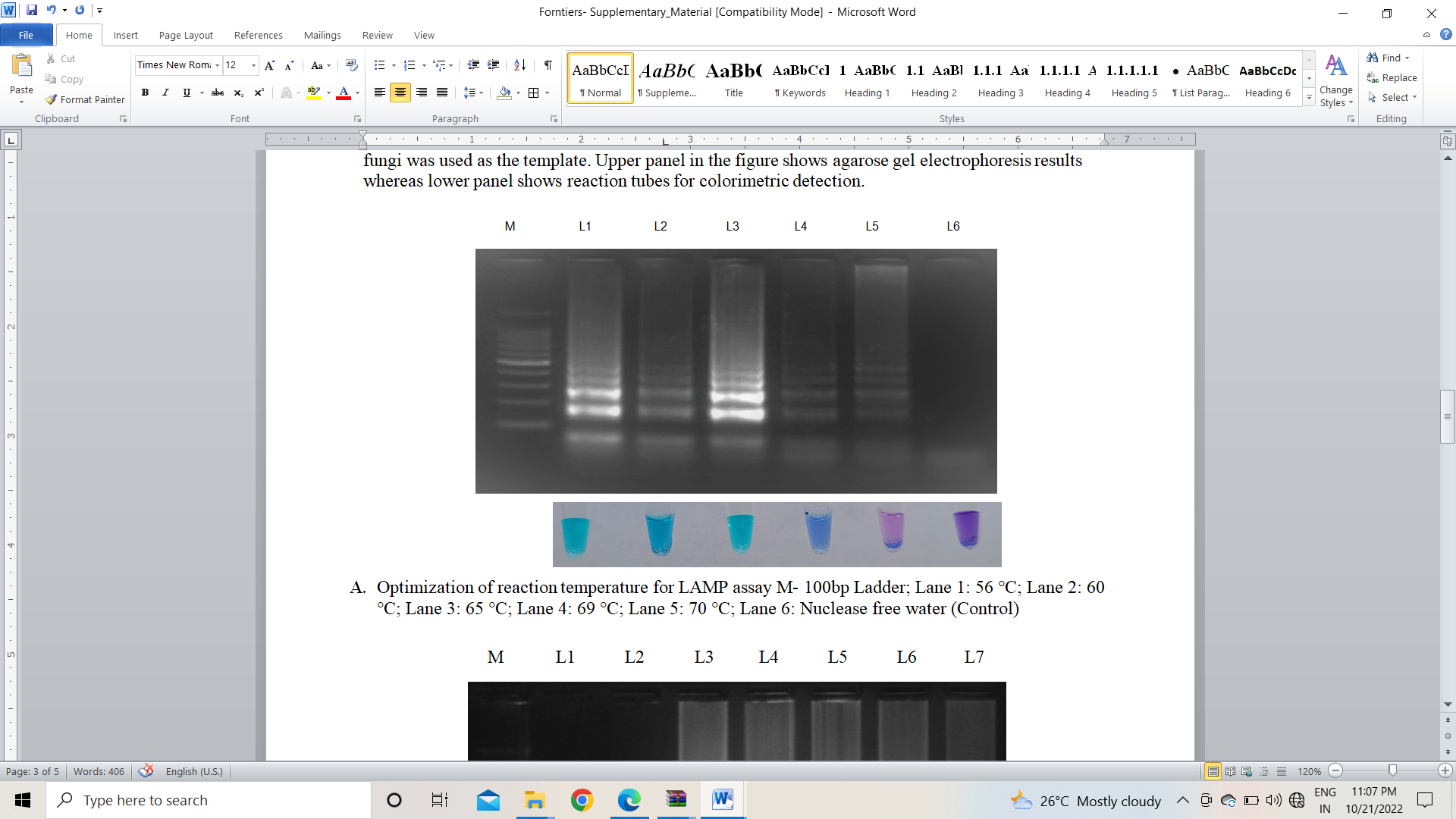 | B. 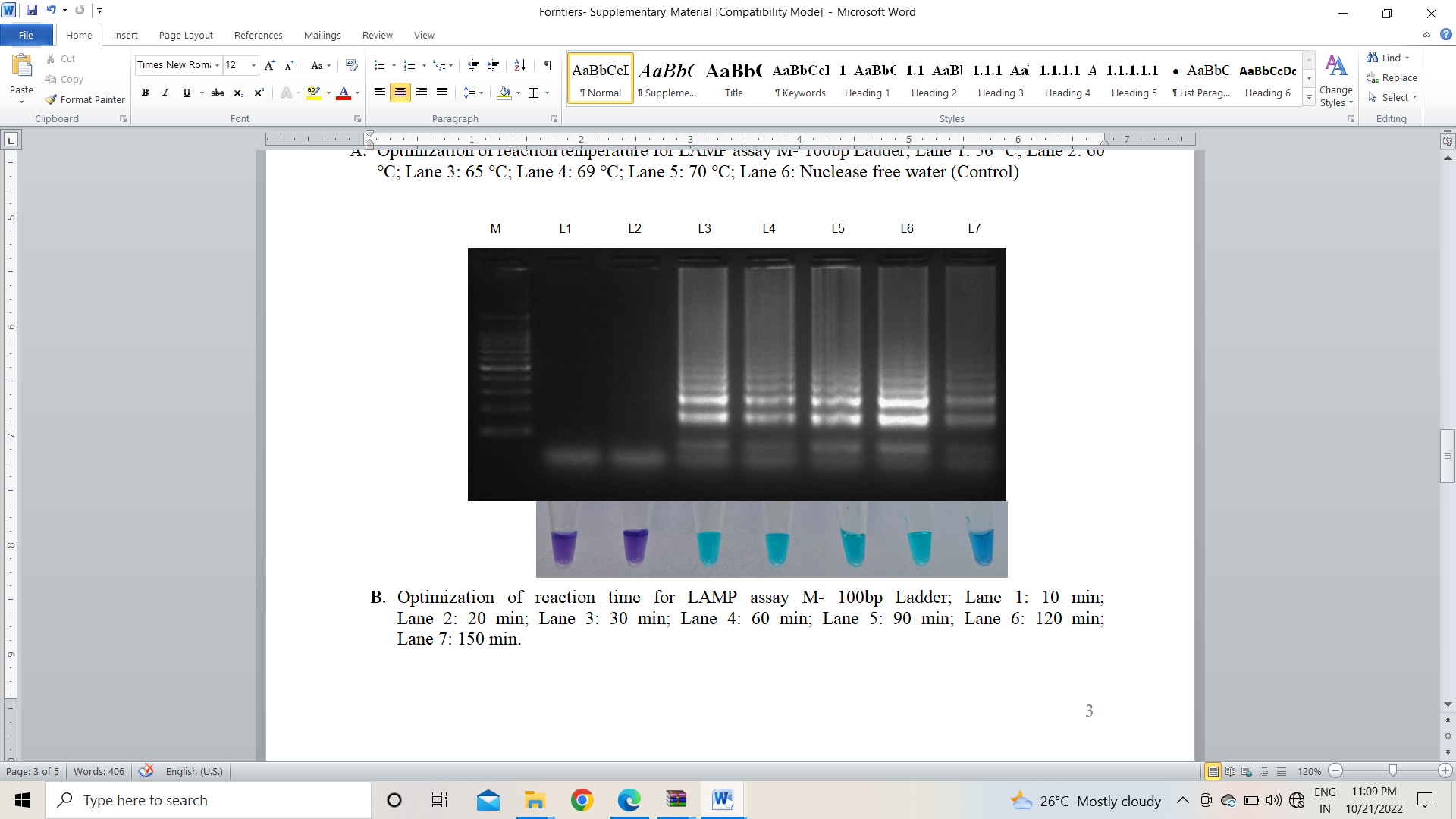 |
| --- | --- |

**Figure S2:** Optimization of LAMP conditions. Bright sky blue colour represents positive reaction while the violet colour indicates the negative reaction. Total DNA isolated from fungi was used as the template. Upper panel in the figure shows agarose gel electrophoresis results whereas lower panel shows reaction tubes for colorimetric detection. M- 100bp Ladder;
**A. R**eaction temperature Lane 1-6: 56 °C, 60 °C, 65 °C, 69 °C, 70 °C, Nuclease free water (Control)
**B.** Reaction time Lane 1-7: 10 min, 20 min, 30 min, 60 min, 90 min, 120 min, 150 min.

| 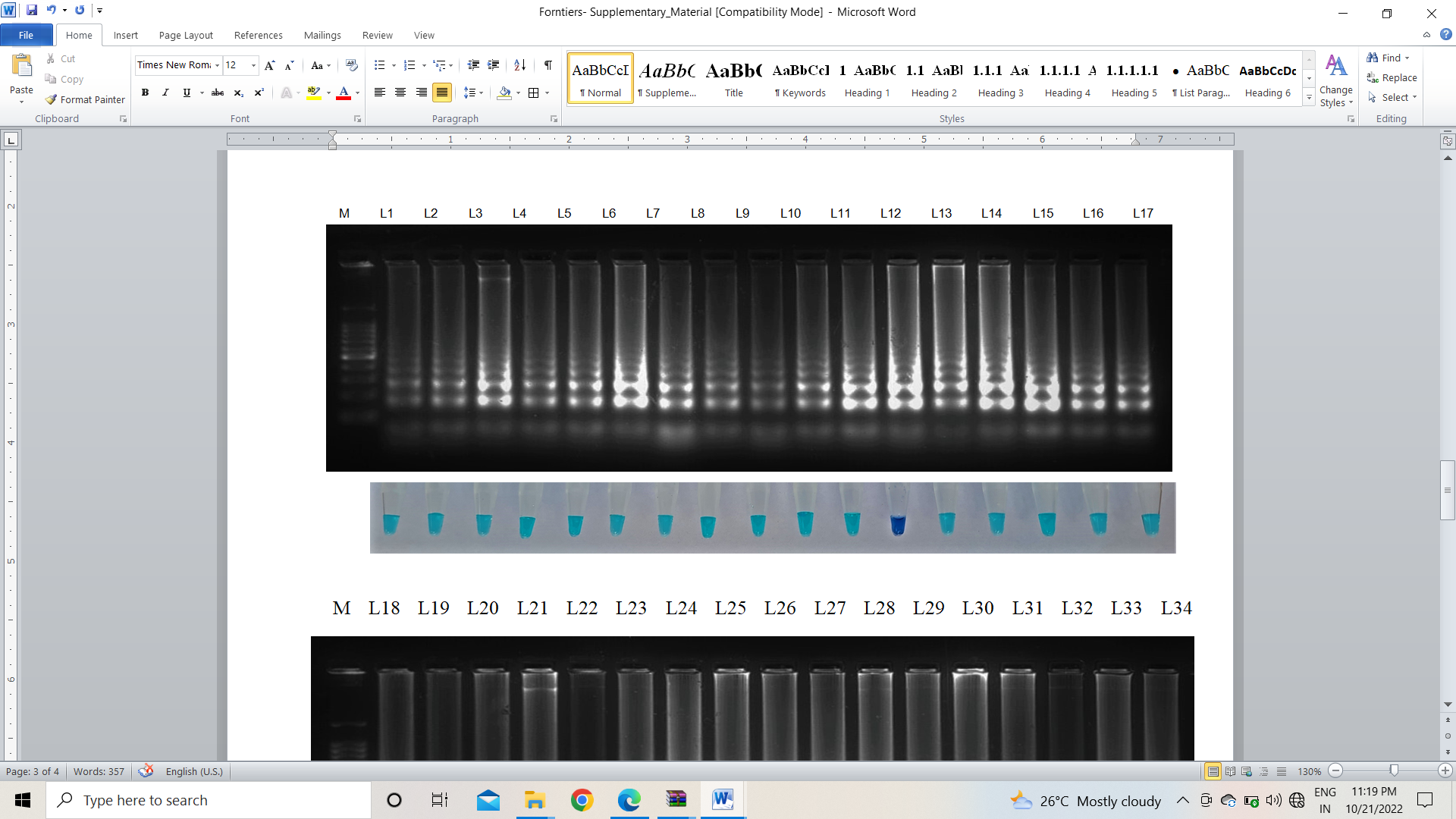 |
| --- |
| 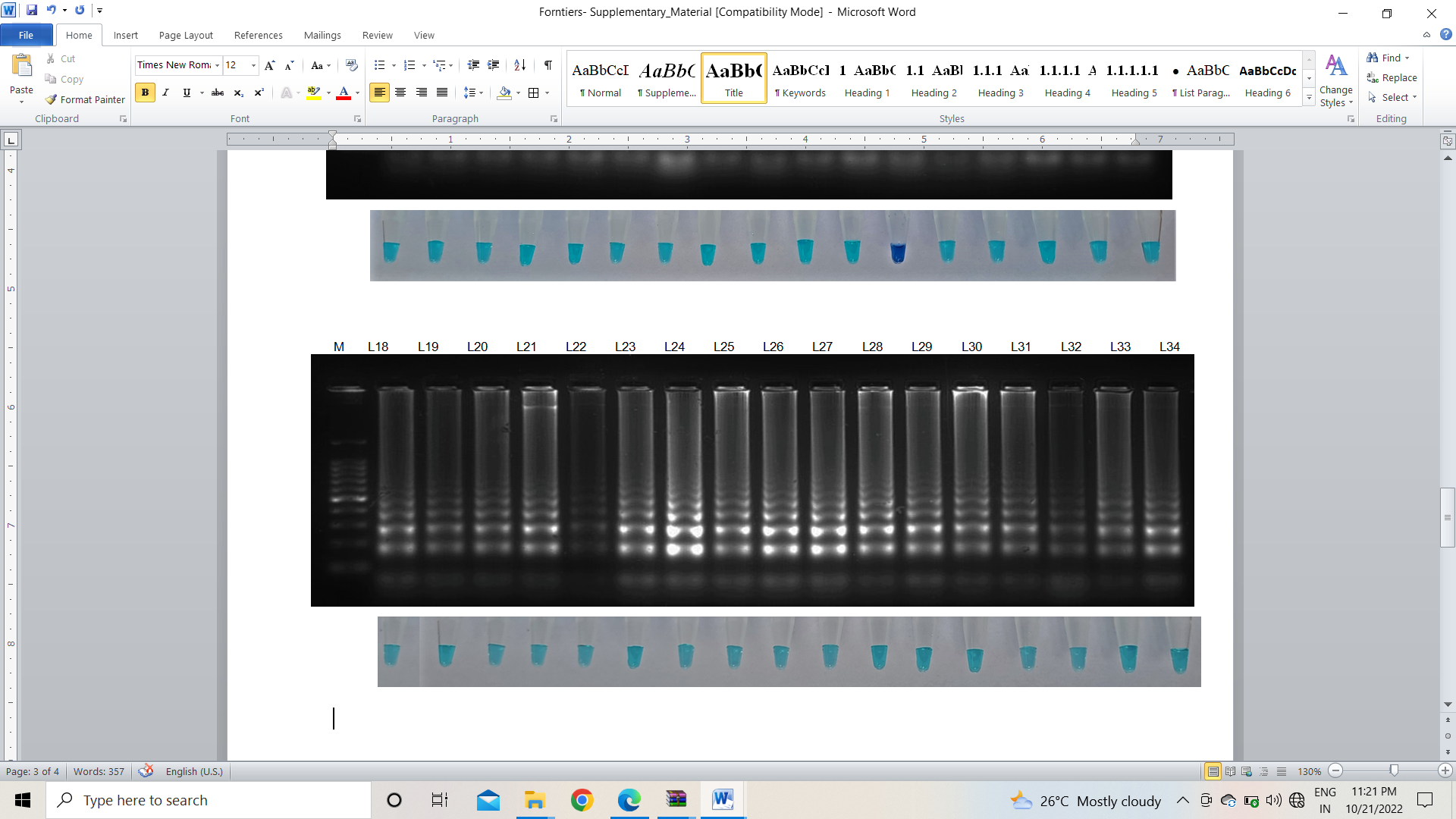 |
| 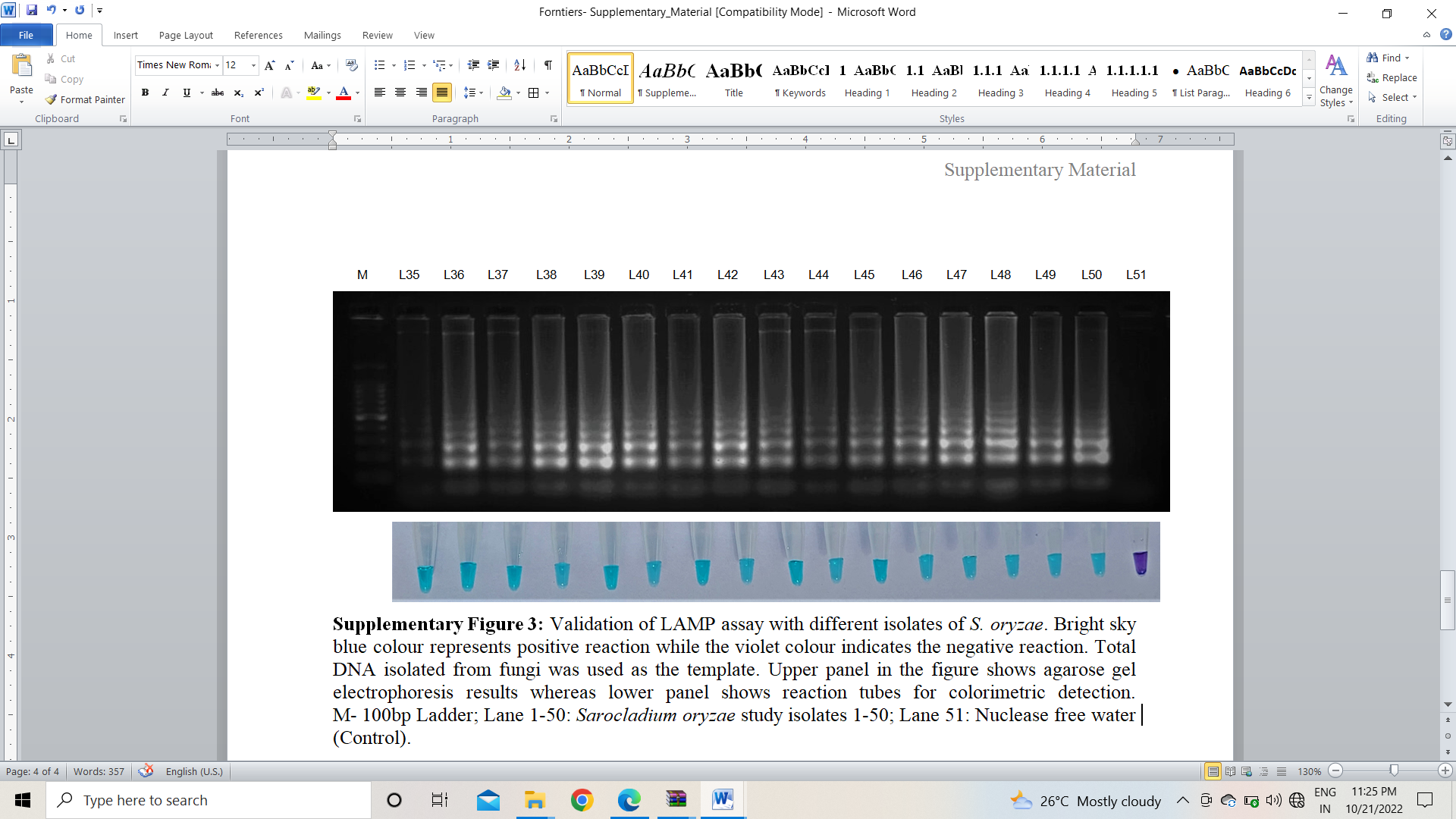 |

**Figure S3:** Validation of LAMP assay with different isolates of *S. oryzae*. Bright sky blue colour represents positive reaction while the violet colour indicates the negative reaction. Total DNA isolated from fungi was used as the template. Upper panel in the figure shows agarose gel electrophoresis results whereas lower panel shows reaction tubes for colorimetric detection.
M- 100bp Ladder; Lane 1-50: *Sarocladium oryzae* study isolates 1-50; Lane 51: Nuclease free water (Control).

**Table S1: Genebank accession number of ITS region of *S. oryzae***

| **S. No.** | **Isolate code** | **Location** | **Accession no.** |
| --- | --- | --- | --- |
| 1. | So 1 | Coimbatore | OM841519 |
| 2. | So 2 | Thirukovilur | OK018129 |
| 3. | So 3 | Vasanthakrishnapuram | OK018130 |
| 4. | So 4 | Coimbatore | OK031107 |
| 5 | So 5 | Chidambaram | OK032120 |
| 6. | So 6 | Emappur | OK018133 |
| 7. | So 7 | Thiruvannamalai | OK018137 |
| 8. | So 8 | Kondathur | OM841478 |
| 9. | So 9 | Coimbatore | OM812089 |
| 10. | So 10 | Thiruvennainallur | OM841481 |
| 11. | So 11 | Mayiladuthurai | OM343181 |
| 12. | So 12 | Kallakurichi | ON084817 |
| 13. | So 13 | Sathyamangalam | OP099854 |
| 14. | So 14 | Someshwarapuram | OM343185 |
| 15. | So 15 | Thirupazhanam | OM841483 |
| 16. | So 16 | Sirkazhi | OM841484 |
| 17. | So 17 | - Papanasam | OM841485 |
| 18. | So 18 | - Puduchatiram | OM919528 |
| 19. | So 19 | - Saliyantoppu | OM841489 |
| 20. | So 20 | - Tanjore | OM991946 |
| 21. | So 21 | - Vadakurangaduthurai | OM841491 |
| 22. | So 22 | - Pudhur, S | OM841497 |
| 23. | So 23 | - Ganapathiagaram | OM841499 |
| 24. | So 24 | - Rayampettai,Tanjore | OM892668 |
| 25. | So 25 | - Aduthurai | OM919508 |
| 26. | So 26 | - Athukudi | ON008305 |
| 27. | So 27 | - Sivapuri, CDM | OM992220 |
| 28. | So 28 | Kallanai,Tanjore | OM919515 |
| 29. | So 29 | - Govindhanallucheri | OM919516 |
| 30. | So 30 | - Thiruvidaimaruthur | ON112283 |
| 31. | So 31 | - Chellapatty, Madurai | OM919518 |
| 32. | So 32 | - Bhavanisagar | ON112364 |
| 33. | So 33 | - Dindugal | ON008316 |
| 34. | So 34 | - Kodikulam, Madurai | ON112988 |
| 35. | So 35 | - Samayapuram | ON115184 |
| 36. | So 36 | - Perambalur | ON115188 |
| 37. | So 37 | - Palaiyur, Thiruvarur | ON115189 |
| 38. | So 38 | - Purathakudi | ON008436 |
| 39. | So 39 | - Aduthurai | ON115190 |
| 40. | So 40 | - Perugamani, Trichy | ON115191 |
| 41. | So 41 | - Kumaramangalam | ON115203 |
| 42. | So 42 | - Perugamani | ON009020 |
| 43. | So 43 | - Orayur | ON009071 |
| 44. | So 44 | - Vayalur | ON115207 |
| 45. | So 45 | - Yethapur, Salem | ON115213 |
| 46. | So 46 | - K. Sathanoor | ON115234 |
| 47. | So 47 | - Maruthandakurichi | OP099852 |
| 48. | So 48 | - Mochakottapalayam | ON009255 |
| 49. | So 49 | - Melur, Madurai | OP377086 |
| 50. | So 50 | - Kulithalai | OP377796 |

**Table S2:** Additional details of designed LAMP primers in this study.

| **Primer name** | **Molecular Weight** | **Extinction Coefficient** | **µg/OD at 260 nm** | **Run Length (bp)** | **Primer Dimer** | **Secondary Structure** | **Binding site** | **Id ^1^**  **(%)** |
| --- | --- | --- | --- | --- | --- | --- | --- | --- |
| SoT-F3 | 6148.1 | 188.4 | 32.6 | 3 | No | [Moderate](http://www.oligoevaluator.com/ShowStructure.jsp?Structure=2_0) | 444-463 | 100 |
| SoT -B3 | 6175.1 | 203.6 | 30.3 | 4 | No | [Very Weak](http://www.oligoevaluator.com/ShowStructure.jsp?Structure=2_0) | 624-643 | 100 |
| SoT -FIP | 12347.1 | 380.4 | 32.5 | 3 | No | [Moderate](http://www.oligoevaluator.com/ShowStructure.jsp?Structure=2_0) | 483-517 | 100 |
| SoT -BIP | 12675.4 | 396.8 | 31.9 | 4 | No | [Weak](http://www.oligoevaluator.com/ShowStructure.jsp?Structure=2_0) | 574-600 | 100 |
| SoT- LF | 6817.5 | 227.1 | 30.0 | 3 | No | None | 484-505 | 100 |
| SoT- LB | 5764.8 | 174.3 | 33.1 | 2 | No | [Strong](http://www.oligoevaluator.com/ShowStructure.jsp?Structure=2_0) | 576-594 | 100 |

^1^ Percentage identity of primer sequence compared to *S. oryzae* genomes available in GenBank using BLAST.
